# Sarcopenia and Its Individual Traits Independently Predict Mortality in Patients on Dialysis: A Systematic Review and Meta‐Analysis

**DOI:** 10.1002/jcsm.70089

**Published:** 2025-10-09

**Authors:** Alice Sabatino, Alessandro Guerra, Kenia Mara Baiocchi de Carvalho, Lilian Cuppari, Juan Jesus Carrero, Peter Stenvinkel, Bengt Lindholm, Carla Maria Avesani

**Affiliations:** ^1^ Division of Renal Medicine, Baxter Novum, Department of Clinical Science, Intervention and Technology Karolinska Institutet Stockholm Sweden; ^2^ Nephrology Unit, Parma University Hospital, and Department of Medicine and Surgery University of Parma Parma Italy; ^3^ Faculty of Health Sciences, Graduate Program in Public Health University of Brasilia Brazil; ^4^ Division of Nephrology Federal University of São Paulo São Paulo Brazil; ^5^ Department of Medical Epidemiology and Biostatistics Karolinska Institutet Stockholm Sweden

**Keywords:** chronic kidney disease, dialysis, mortality, muscle mass, muscle strength, sarcopenia, systematic review

## Abstract

**Background:**

Loss of muscle mass, muscle weakness and the combination of the two are commonly observed in patients on dialysis and may have a negative impact on their survival. In this systematic review and meta‐analysis (MA), we evaluated the consistency and strength of the association between mortality risk and the presence of low muscle mass with adequate muscle strength, low muscle strength with adequate muscle mass and sarcopenia (low muscle mass and strength combined) in patients on dialysis. Ultimately, we aimed to grade which of these three conditions had the strongest association with increased mortality risk in this vulnerable group of patients.

**Methods:**

We searched for studies published until 31 January 2024 that evaluated sarcopenia and its individual components in patients aged > 18 years on dialysis (haemodialysis and peritoneal dialysis) and that had a mean follow‐up for mortality for ≥ 12 months. Included studies had to enable the evaluation of sarcopenia traits to identify patients with low muscle mass and adequate muscle strength, low muscle strength with adequate muscle mass, and sarcopenia. A systematic search was conducted in MEDLINE (by PubMed), Embase, Web of Science, Lilacs and grey literature (i.e., Google Scholar and ProQuest). We estimated consistency in the association between sarcopenia traits and death using random effects MA and reporting hazard ratio (HR) with a 95% confidence interval (95% CI).

**Results:**

The electronic search retrieved 5712 records. After removing duplicated records and those that did not meet the eligibility criteria, 19 studies were included in the qualitative synthesis (4281 participants) and 17 studies (4024 participants) with extractable data for the MA. The MA showed low heterogeneity in the association between muscle parameters and sarcopenia with the risk of death: *low muscle mass with adequate muscle strength,* HR: 1.49; 95% CI: 1.04 to 2.13; *I* ^2^ = 0%; *low muscle strength with adequate muscle mass,* HR: 1.82; 95% CI: 1.38 to 2.41; *I* ^2^ = 0%; and *sarcopenia,* HR: 2.02; 95% CI: 1.61 to 2.54; *I* ^2^ = 40%.

**Conclusion:**

Although being statistically significant, the association between low muscle mass and mortality seems to be less strong than the association between low muscle strength and mortality in patients on dialysis. Those presenting with a combination of the two traits, that is, sarcopenia, showed the highest risk of dying prematurely.

## 1 Introduction

Patients with chronic kidney disease (CKD) are exposed to conditions intrinsic to their disease and to various catabolic factors including the dialysis procedure that collectively increase protein degradation, decrease protein synthesis, and result in negative protein balance [1]. The latter, if maintained, leads to muscle abnormalities including a decrease in muscle mass, weakening of muscle strength and impaired physical function [1, 2]. The concomitant occurrence of low muscle strength and low muscle mass is the operational definition of sarcopenia according to the revised consensus of the European Working Group for Sarcopenia in Older People (EWGSOP 2) and the Global Leadership Initiative in Sarcopenia (GLIS) [3, 4].

After the publication of the first consensus report on sarcopenia diagnosis by the EWGSOP in 2010 [5], much attention has been given to muscle abnormalities in clinical conditions with increased protein catabolism, such as cancer, congestive heart failure, HIV and CKD [1, 6, 7, 8]. Of note, sarcopenia can be present even in obese individuals (i.e., obese or secondary sarcopenia) and in young adults exposed to catabolic conditions [9, 10]. A subsequent 2019 EWGSOP consensus report [3] defined sarcopenia as a progressive and generalized skeletal muscle disorder that is associated with an increased likelihood of adverse outcomes including falls, fractures, physical disability and mortality, underlining the importance of testing for sarcopenia in different clinical settings. In addition, sarcopenia has been recognized as a disease entity with its own ICD‐10‐CM code (M62.84), allowing treatment and reimbursement from medical insurances [3].

Because of heterogeneity in methods and operating definitions, studies that evaluated sarcopenia in patients with CKD show big variations in prevalence according to an MA of 140 studies in patients with CKD in all disease stages: The prevalence of sarcopenia varied from 11% to 30% with higher prevalence in CKD patients on dialysis (26.2%; 95% confidence interval [CI]: 16.6 to 37.1) than in those not undergoing dialysis (3.0%, 95% CI: 0 to 11.1) [11]. This wide variation may have led to therapeutic nihilism regarding the usefulness of screening and diagnosing this condition. However, regardless of definitions, sarcopenia has been repeatedly associated with worse quality of life [12], higher hospitalization rates [12] and increased mortality [13, 14] in patients with CKD in all disease stages [1].

Studies comparing the prognostic value of individual sarcopenia traits in these patients suggest stronger independent associations for markers of muscle strength over muscle mass [15, 16], which aligns with the recommendation of EWGSOP 2 for the geriatric population of prioritising muscle strength screening over muscle mass [3]. However, to the best of our knowledge, available systematic reviews and meta‐analyses did not evaluate the prognostic impact of a single muscle abnormality, namely low muscle mass with adequate muscle strength or low muscle strength with adequate muscle mass. Understanding the consistency of these associations may help reconcile disagreements on the value of monitoring muscle mass and strength in routine clinical practice. Thus, we performed a systematic review and MA to evaluate the associations of low muscle mass or low muscle strength, or both conditions (i.e., sarcopenia), with the risk of death of patients on dialysis.

## 2 Methods

### 2.1 Protocol and Registration

This systematic review and MA was conducted according to the Preferred Reporting Items for Systematic Reviews and Meta‐Analyses (PRISMA 2020) guidelines for observational studies [17] and was registered in the International Prospective Register of Systematic Reviews (PROSPERO; registration number CRD42021229705).

### 2.2 Eligibility Criteria

Eligibility to be included in our study was based on the Population, Exposition, Comparison, Outcomes and Study design (PECOS), as described in Table S1 [18]. We included longitudinal observational studies that had a mean or median follow‐up for survival of at least 1 year, that assessed sarcopenia using the operational criteria (low muscle strength and low muscle mass combined) according to the revised EWGSOP 2 [3] and/or that reported results for each muscle abnormality (i.e., low muscle mass with adequate muscle strength, low muscle strength with adequate muscle mass and low muscle mass and muscle strength combined [named sarcopenia]). We retained studies that comprised patients aged 18 years and older on maintenance dialysis (haemodialysis [HD] and peritoneal dialysis [PD]), both incident and prevalent patients. We excluded studies where the population of interest included mainly pregnant women, patients with cancer, acquired immunodeficiency syndrome, Alzheimer's and Parkinson's diseases, chronic obstructive pulmonary disease, acute kidney disease, kidney transplanted and bedridden patients. Studies that assessed muscle strength by questionnaires and muscle mass by serum creatinine were not retained. Letters to editors, narrative reviews, clinical cases and case reports were not considered. No restrictions were applied on publication date and status of the publication. Studies not including an abstract in English were not included.

### 2.3 Information Sources and Search Strategies

The search strategy was revised by two external researchers experienced with systematic review following the checklist of the Peer Review of Electronic Search Strategies (PRESS checklist) [19]. Once the search strategy was defined, a systematic search of the following databases was conducted to identify relevant studies: Medline (by PubMed), Embase, Web of Science and Lilacs. Grey literature search was performed in Google Scholar and in the ProQuest Dissertation and Theses Global (PQDT) database. The search was conducted on 18 January 2021 and updated on 31 January 2024. Free text words and MeSH terms were used during the search strategies and are described in Table S2. The Google search was limited to the first 200 most relevant articles.

### 2.4 Study Selection

Zotero Software (Corporation for Digital Scholarship, Virginia, USA) [20] and Rayyan QCRI software (Qatar Computing Research Institute, Doha, Qatar) [21] were used to remove duplicate references and for the screening procedure. Study selection was undertaken independently in two phases by two reviewers. First, articles were screened according to their titles and abstracts, and studies that did not meet the eligibility criteria were eliminated. Then, articles were read in full, and those eligible were selected for review. The disagreements were resolved by consensus and, when necessary, by the participation of a third author. The reasons for the exclusion of studies at this stage were duly registered.

### 2.5 Data Extraction and Quality Assessment

Data were extracted by one author and revised by two authors. The following data were collected: authors, year of publication, country, dialysis therapy (HD/PD), follow‐up (months), sample size and sex distribution (percentage of women), age (mean or median), method and cutoff points to evaluate muscle mass and muscle strength at baseline; sarcopenia diagnosis, sarcopenia percentage, proportion of deaths, mortality risk analysis, confounding variables used in adjusted analysis and additional results. In case of unavailable data, the corresponding authors of the studies were contacted for clarification. If no answer was obtained, the article was not considered for MA.

The assessment for the methodological quality of included studies was performed using The Critical Appraisal Tool for cohort studies as recommended by the Joanna Briggs Institute (JBI) [22]. The purpose of the JBI critical appraisal tool is to assess the methodological aspects of a study and to determine the extent to which a study has addressed the possibility of bias in its design, conduct and analysis. This appraisal consists of a checklist containing 11 questions answerable by “yes,” “no,” “unclear,” or “not applicable.” The higher the number of “yes” answers obtained, the greater the methodological rigour of the study and the lower the risk of bias. Thus, in this study, the risk of bias was considered low when all items were answered “yes,” while it was considered high when at least one item was answered “no.” No scores were assigned, and results for each question were expressed as the frequency for each classification. This process was performed independently by two authors. The disagreements were resolved by a third reviewer.

For each method of assessment, confidence in the evidence included in the MA (risk of bias, consistency, directness and precision) was assessed using the Grading of Recommendations Assessment, Development and Evaluation methodology (GRADE) [23]. The GRADE System is comprised of eight items that define the quality of evidence of the following evaluated outcomes: risk of bias, inconsistency, indirect evidence, imprecision, publication bias, effect magnitude, dose–response gradient and evaluation of the effect of confounding variables. The final evidence score can be classified as high (≥ 4 points), moderate (3 points), low (2 points) or very low (1 point). Because of the observational design of the included studies, all GRADE ratings started as low. Evidence certainty was upgraded based on the evidence of a large and strong association with mortality risk even after adjustment for plausible covariates.

### 2.6 Data Synthesis and Analysis

Data analysis was performed using SPSS version 29 (IBM SPSS Statistics Inc. Chicago, IL, USA). Statistical heterogeneity was assessed using the *I* ^2^. The magnitude of data inconsistency was classified according to the following parameters: 0%–40%: might not be important; 30%–60%: may represent moderate heterogeneity; 50%–90%: may represent substantial heterogeneity; and 75%–100%: considerable heterogeneity. Additionally, the Galbraith graph was used to identify outlier studies. All studies that showed standardized effect values in the Galbraith plot between values 2 and −2 were not considered outliers. The groups were evaluated according to this statistical criterion [24].

The hazard ratio association measure and 95% CIs were estimated for each outcome through random effects MA using the DerSimonian‐Laird method and shown in the forest plot. Publication bias was estimated using Egger's test with 10% statistical significance and inspection of the funnel when more than 10 studies were included in the MA [25, 26]. Subgroup analyses were performed by age (papers with mean age ≥ 60 vs. < 60 years), dialysis vintage (incident vs. prevalent patients) and sex (female prevalence > 50% and < 50%) using the same methodology as described for the main analysis. A sensitivity analysis was performed including only the papers that had information on both low muscle mass and low muscle strength.

## 3 Results

### 3.1 Study Selection

**FIGURE 1:**
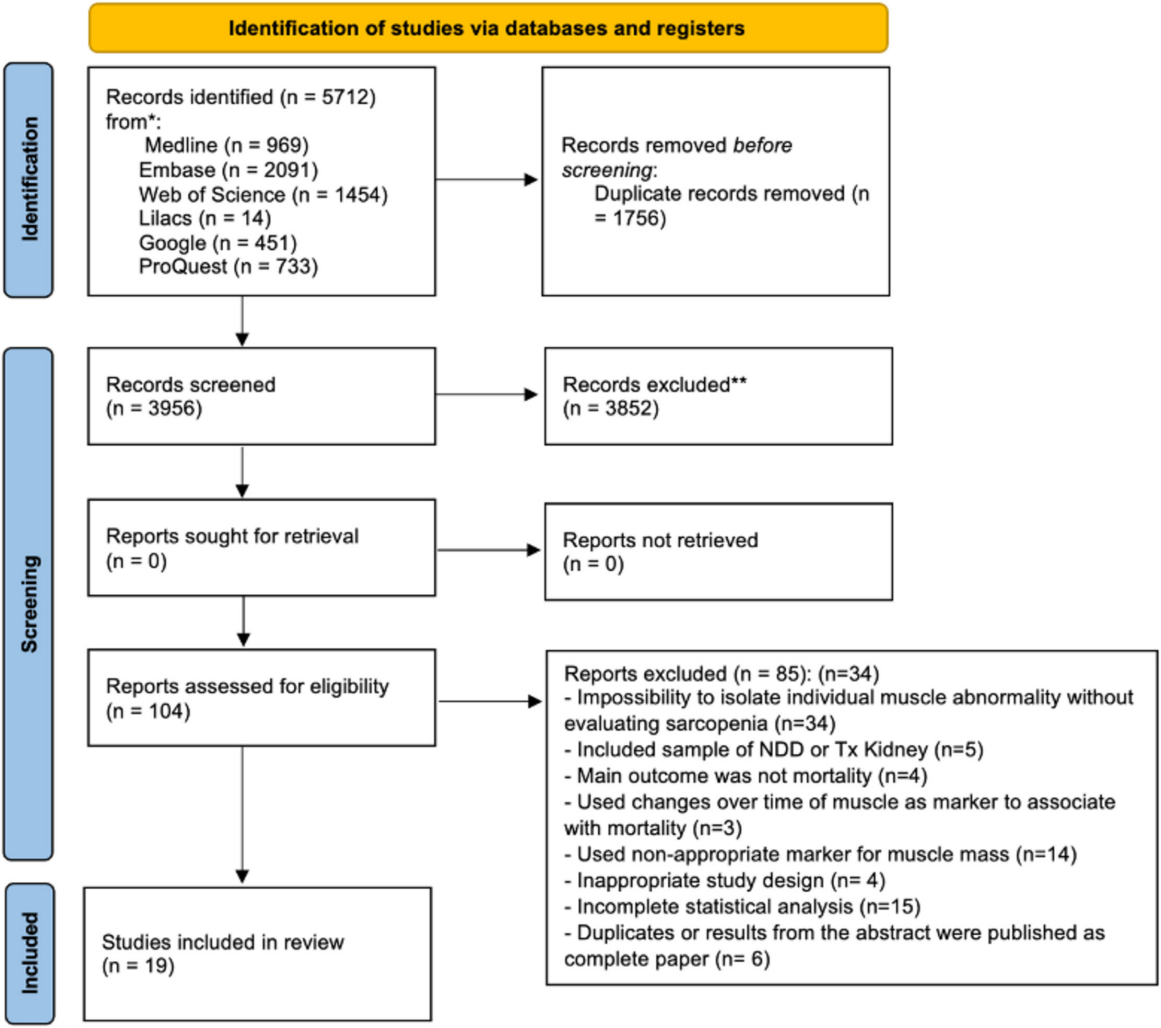
Flow diagram of the literature search and selection criteria.

As described in Figure 1, the electronic search retrieved 5712 records. After removing duplicated records and those that did not meet the eligibility criteria, 19 studies were included in the qualitative synthesis (4281 participants) and 17 studies (4024 participants) with extractable data for the MA.

### 3.2 Study Characteristics

The characteristics of the studies according to the three measurements of exposure, that is, muscle mass, muscle strength and sarcopenia, are shown in Table 1 (for details of each study separately, see Tables S3A, 3B and C). We identified studies from 4 continents, except Africa. The studies came mostly from China [27, 28, 29, 30], Japan [31, 32, 33] and Brazil [12, 34, 35]. The mean and median age of participants was > 50 years, except for one study [29]. All of them included both sexes. In general, studies included patients on HD and only one study included patients on PD [27]. The methods most used to evaluate muscle mass were bioelectrical impedance analysis (BIA) or bioelectrical impedance spectroscopy (BIS) [15, 28, 29, 30, 36, 37, 38, 39, 40], and dual energy x‐ray absorptiometry (DXA) [32, 33, 34, 41]. Other applied methods were body composition equations [12, 27], creatinine index [31, 42], calf circumference [35] and ultrasound [43]. Muscle strength was evaluated by handgrip strength (HGS) in most studies [12, 15, 27, 28, 29, 30, 31, 32, 33, 34, 35, 36, 37, 38, 39, 40, 41, 43] and only one study assessed muscle strength of the lower limbs by maximal voluntary force [42]. Studies had different follow‐up times, with a minimum of 12 months [29] and a maximum of 90 months [33].

**TABLE 1:** Summary of study characteristics and main results separated by measurement of interest in patients on dialysis.

Characteristics	Articles assessing		Articles assessing		Articles assessing	
	Muscle mass		Muscle strength		Sarcopenia	
	*n*	%	*n*	%	*n*	%
**Geographic region**						
America	1	17%	1	12%	4	21%
Africa	0	0	0	0%	0	0%
Asia	3	50%	3	38%	10	53%
Europe	2	33%	3	38%	4	21%
Oceania	0	0	1	12%	1	5%
**Sample size**						
≤ 500	5	83%	7	88%	17	89%
> 500	1	17%	1	12%	2	11%
**Dialysis modality**						
HD	5	83%	7	88%	18	95%
PD	1	17%	1	12%	1	5
Sex**						
**>** 50% male sex	6	100%	8	100%	17	89%
≤ 50% male sex	0	0%	0	0%	2	11%
Age*						
< 60 years	3	50%	5	63%	8	47%
≥ 60 years	3	50%	3	37%	9	53%
Prevalence of muscle disorder**						
≤ 10%	0	0%	0	0	2	11%
10–25%	4	80%	6	86%	3	17%
> 25%	1	20%	1	14%	13	72%
**Prevalence of mortality**						
≤ 10%	0	0%	0	0%	2	11%
10–25%	2	33%	1	12%	4	21%
> 25%	4	67%	7	88%	13	68%

Abbreviations: HD, haemodialysis; PD, peritoneal dialysis.

Muscle mass: ** one study missing data.

Muscle strength: ** one study missing data.

Sarcopenia: * two studies missing data; ** one study missing data.

### 3.3 Risk of Bias and Quality of Evidence

**FIGURE 2:**
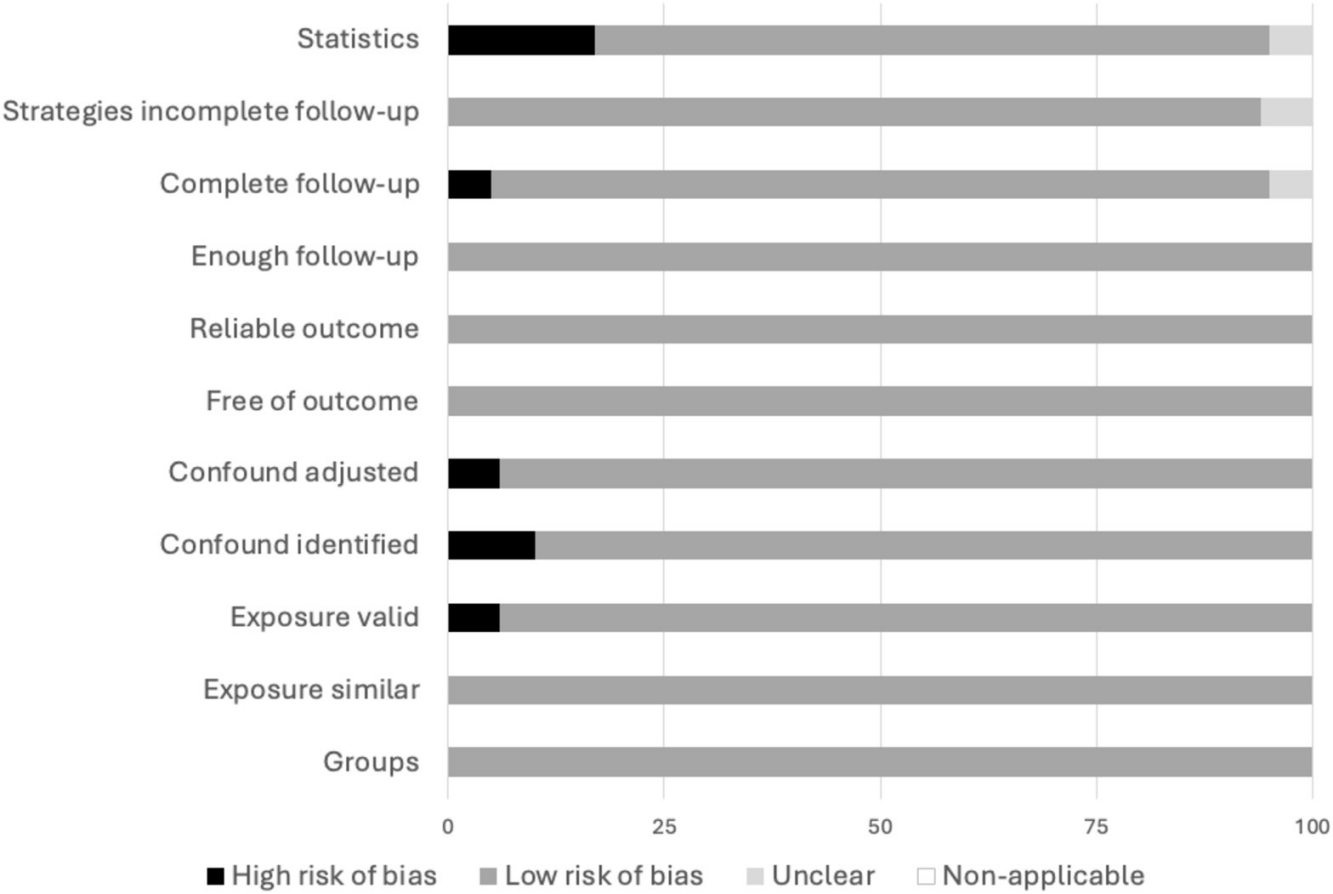
Risk of bias by each component of assessment.

Thirteen out of 19 studies were considered to have a low risk of bias in all assessed parameters. Three studies had unclear risk because of lack of information [32, 36, 40], while one study had only one assessment item with a high risk of bias [27], and the remaining two studies had two or more items with a high risk of bias [29, 37] (Figure S1). Figure 2 describes the risk of bias by the components of assessment. All studies reported the length of follow‐up and used a reliable way to assess the outcome of interest. Moreover, all patients were free of the outcome at study enrolment and the exposure (low muscle mass and/or low muscle strength) was measured in a similar way for all patients independently of the outcome. Information regarding the completion of the follow‐up for all subjects enrolled was absent in 5% of the studies and unclear in 5%. Only 5% of the studies were unclear regarding strategies to deal with patients that did not complete follow‐up and hence were censored without reaching the outcome. Finally, 15.8% of studies did not perform appropriate statistical analysis, while in 5% it was unclear.

The overall GRADE for studies evaluating low muscle mass and low muscle strength was scored as very low because of the small sample size and low number of studies, which did not allow for the evaluation of publication bias. It was considered moderate for studies investigating sarcopenia due to the stronger association and lack of publication bias (Table S4).

### 3.4 Outcomes

#### 3.4.1 Low Muscle Mass With Adequate Muscle Strength and Mortality Risk

Six studies [12, 27, 31, 36, 41, 43] (1620 participants) were included in the systematic review, comprising five studies with patients on HD [12, 31, 36, 41, 43] and one study on PD [27]. The studies were published from 2014 [41] to 2024 [43]. The percentage of female patients varied from 30% [43] to 47.6% [27]. Two studies included incident patients [27, 41], and the remaining four studies included prevalent patients [12, 31, 36, 43]. The mean (or median) age varied from 53 [41] to 70.6 years [12], the frequency of patients with low muscle mass and adequate muscle strength at baseline ranged from 12.5% [43] to 40.4% [27], and the median follow‐up time for mortality varied from 17.5 [12] to 51.6 months [36].

**FIGURE 3:**
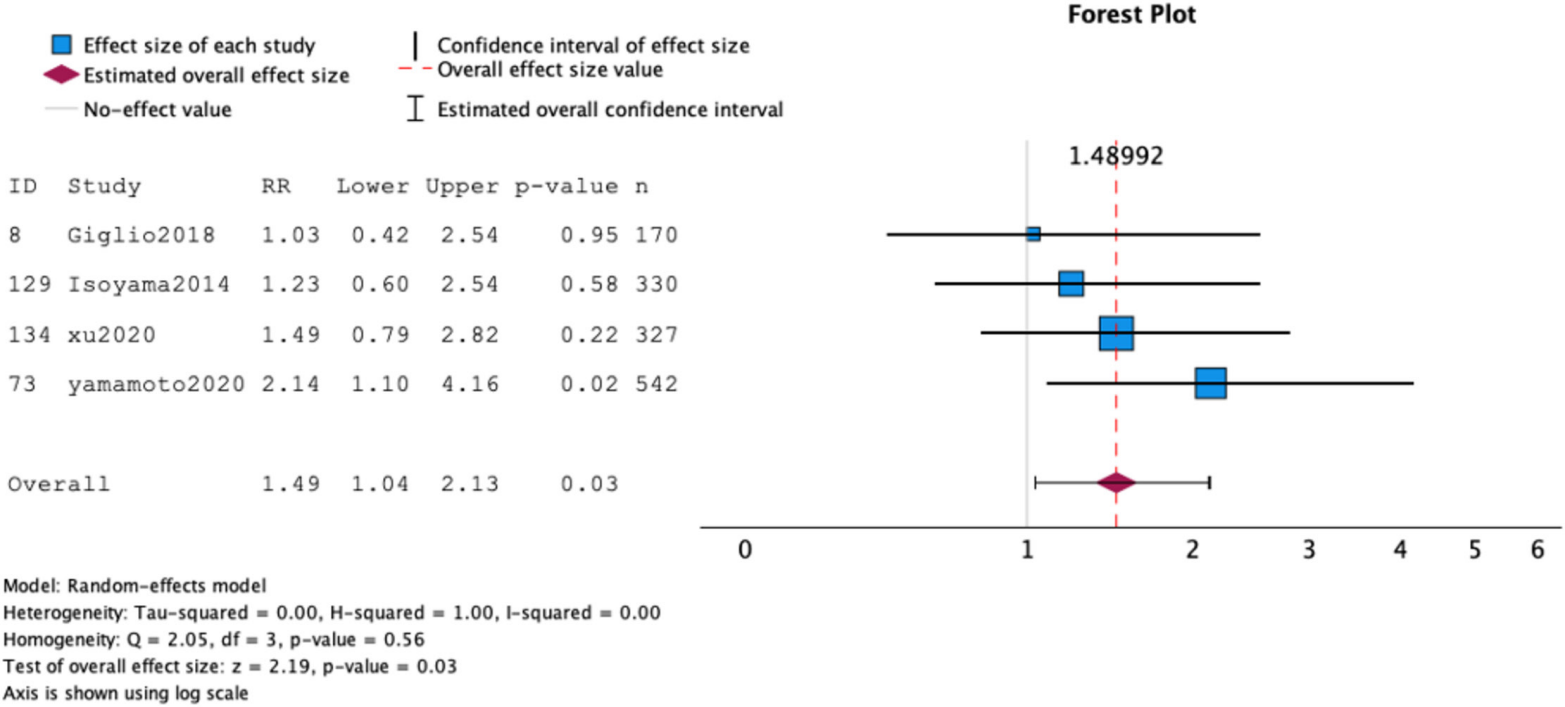
Meta‐analysis for studies assessing low muscle mass with adequate muscle strength and its association with mortality on prevalent patients on dialysis.

Four studies [12, 27, 31, 41] (1369 participants) were included in the MA (Figure 3 **)**. Results show that low muscle mass with adequate muscle strength was associated with an increased risk of mortality with low heterogeneity (HR: 1.49; 95% CI: 1.04 to 2.13; *I* ^2^ = 0.00%) (Figure 3). Because of the low number of studies, it was not possible to evaluate the risk of publication bias.

#### 3.4.2 Low Muscle Strength With Adequate Muscle Mass and Mortality Risk

Eight studies [12, 27, 31, 36, 39, 41, 42, 43] (1874 participants) were included in the systematic review, of which one included patients on PD [27], and the remaining included only patients on HD [12, 27, 31, 36, 39, 41, 43]. The studies were published from 2014 [41] to 2024 [43]. The percentage of female patients varied from 30% [43] to 47.6% [27]. Mean (or median) age varied from 53 [41] to 71 years [39], and the frequency of patients with low muscle strength and adequate muscle mass ranged from 13% [39] to 27.6% [12]. The median (or mean) follow‐up time for mortality varied from 17.5 [12] to 72 months [39].

**FIGURE 4:**
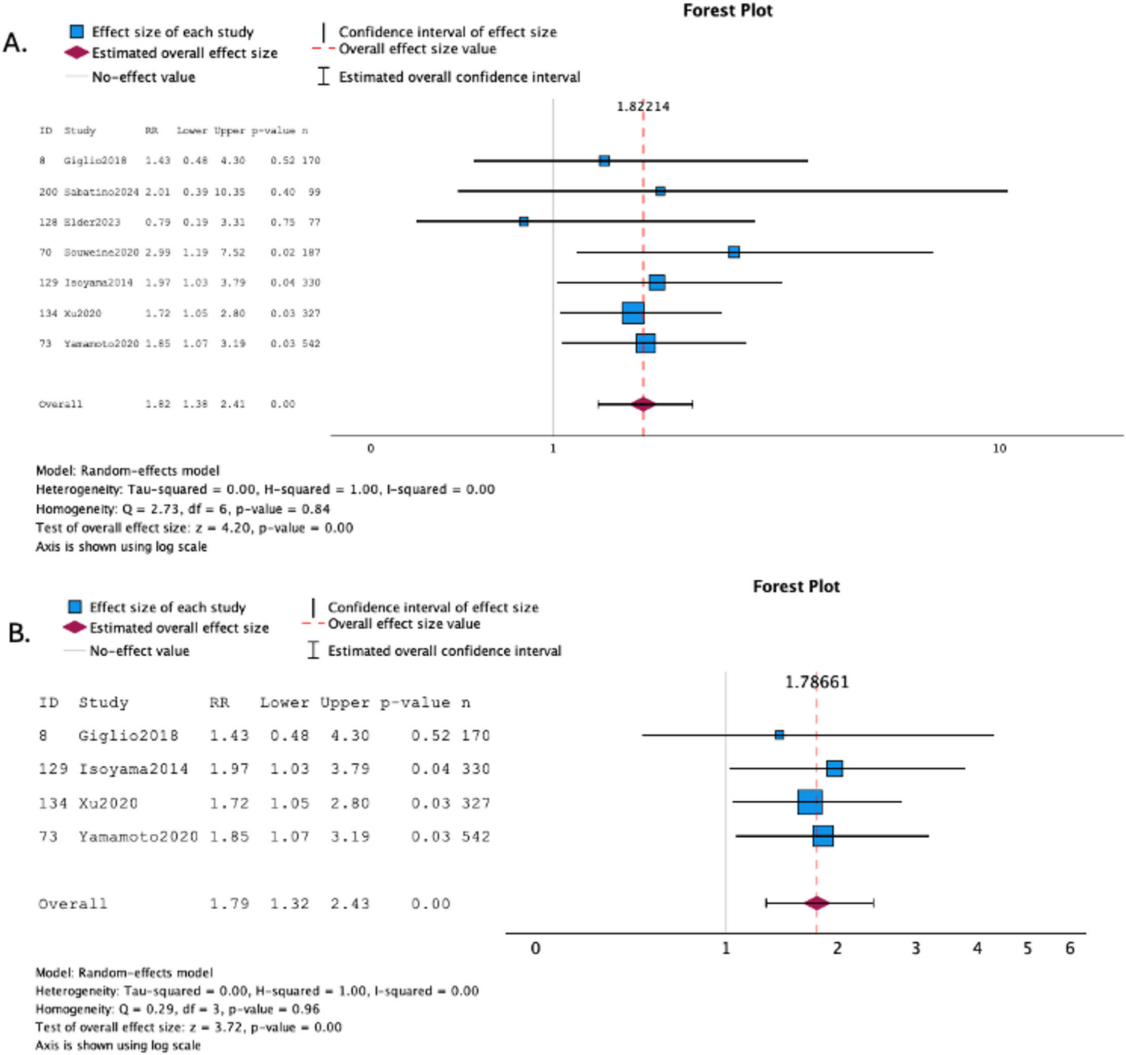
(A–B). Figure 4A. Meta‐analysis for studies assessing low muscle strength with adequate muscle mass and its association with mortality in prevalent patients on dialysis. Figure 4B. Meta‐analysis for studies assessing low muscle strength with adequate muscle mass that were also included in the meta‐analysis for studies assessing low muscle mass with adequate muscle strength and its association with mortality in prevalent patients on dialysis.

Seven studies [12, 27, 31, 39, 41, 42, 43] (1732 participants) were included in the MA (Figure 4A). Results showed that low muscle strength with adequate muscle mass was associated with an increased risk of death with low heterogeneity (HR: 1.82; 95% CI: 1.38 to 2.41; *I* ^2^ = 0.00%) (Figure 4A). Because of the low number of studies included, it was not possible to evaluate publication bias. In the sensitivity analysis, the same four studies included for the low muscle mass analysis also had information on low muscle strength [12, 27, 31, 41] (1369 participants) and were included in the MA (Figure 4B). The association between low muscle strength remained similar to the first analysis with low heterogeneity (HR: 1.79; 95% CI: 1.32 to 2.43; *I* ^2^ = 0.00% and *p* < 0.001) (Figure 4B).

#### 3.4.3 Sarcopenia and Mortality Risk

Sarcopenia (i.e., the simultaneous presence of low muscle strength and low muscle mass) was evaluated in the studies evaluating low muscle and low muscle strength separately and in another three studies that evaluated the role of sarcopenia, meaning a total of 19 studies [12, 15, 27, 28, 29, 30, 31, 32, 33, 34, 35, 36, 37, 38, 39, 40, 41, 42, 43] (4281 participants) published from 2014 [41] to 2024 [43]. From these, only one of the studies included patients on PD [27], and the remaining studies included patients on HD [12, 15, 27, 28, 29, 30, 31, 32, 33, 34, 35, 36, 37, 38, 39, 40, 41, 42, 43]. The percentage of female patients varied from 30% [43] to 52.6% [28], the mean (or median) age varied from 49.4 [29] to 81.9 years [40], the frequency of patients with sarcopenia at baseline ranged from 3.9% [15] to 42.9% [39], and the median follow‐up time for mortality varied from 12 [29] to 90 months [33].

**FIGURE 5:**
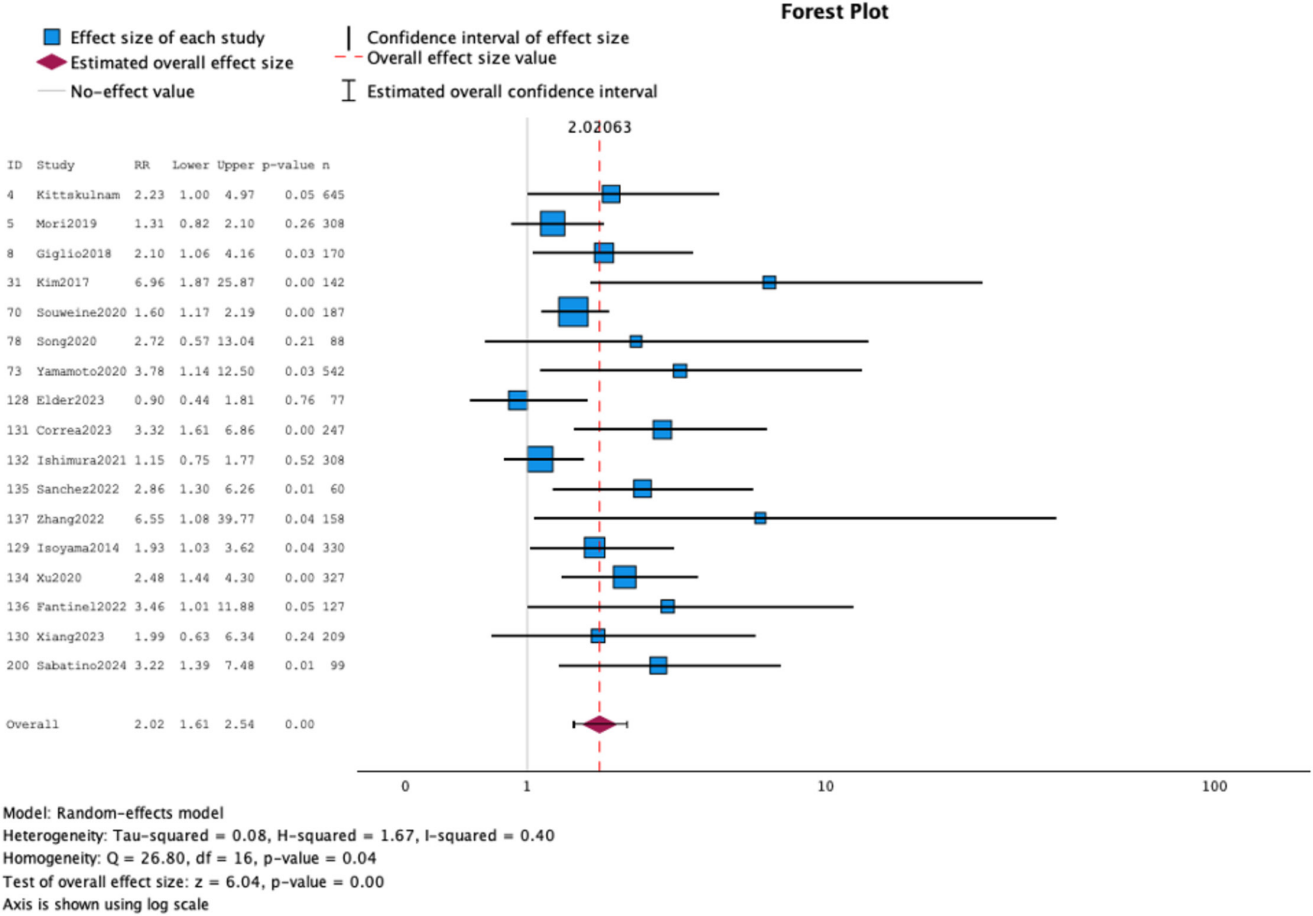
Meta‐analysis for studies assessing confirmed sarcopenia and its association with mortality in prevalent patients on dialysis.

In the MA of 17 studies (4024 participants) [12, 15, 27, 28, 30, 31, 32, 33, 34, 35, 36, 38, 39, 40, 41, 42, 43], the presence of sarcopenia was associated with the risk of death with low heterogeneity (HR: 2.02; 95% CI: 1.61 to 2.54; *I* ^2^ = 40.0%) (Figure 5). After visual inspection of Galbraith's plot (Figure S2), no important outliers were identified. Egger's test supported the absence of publication bias (*p* = 0.895), as shown in the optimal funnel plot (Figure S3).

### 3.5 Subgroup Analyses

#### 3.5.1 Incident Versus Prevalent Patients

When analyzing whether there was a difference in the effect of low muscle mass with adequate muscle strength on mortality in relation to dialysis vintage, the meta‐analyses included two studies per group and results were not significant (Figure S4). In the meta‐analyses evaluating the effect of low muscle strength with adequate muscle strength, the MA included two studies in the incident group [27, 41] and 5 studies in the prevalent group [12, 31, 39, 42, 43]. The increased risk of mortality with low muscle strength with adequate muscle mass was consistent in both groups of patients (incident vs. prevalent patients): HR: 1.80 (1.22–2.67), *p* < 0.001 and 1.84 (1.23–2.75), *p* < 0.001, respectively, with no difference between groups (*p* = 0.95) (Figure S5). In the case of confirmed sarcopenia, two studies were included in the incident group [27, 41], and the remaining 15 studies [12, 15, 28, 31, 32, 33, 34, 35, 36, 38, 39, 40, 42, 43] were included in the prevalent group. In both groups, the effect on mortality was significant: HR incident 2.23 (1.47–3.37), *p* < 0.001 versus HR prevalent 1.93 (1.43–2.60), *p* < 0.001, with no difference in homogeneity between subgroups (*p* = 0.58) (Figure S6).

#### 3.5.2 Patients < 60 Years of Age Versus ≥ 60 Years of Age

We also performed subgroup analyses based on the mean (or median) age of the investigated patients. In the case of low muscle mass with adequate muscle strength and low muscle strength with adequate muscle mass, the studies were divided equally as in the previous subgroup analyses with the same results. For confirmed sarcopenia, the mean (or median) age was below 60 years in 7 studies [15, 27, 28, 30, 32, 36, 41], while in 8 studies, it was above 60 years [12, 31, 34, 38, 39, 40, 42, 43]; two studies were not included because they did not provide the mean (or median) age for the entire cohort [33, 35]. Results from the meta‐analyses showed an effect of sarcopenia on mortality for both groups of patients (HR < 60 years: 2.15 [1.42–3.25], *p* < 0.001 vs. HR ≥ 60 years: 2.11 [1.51–2.96], *p* < 0.001) with no difference in homogeneity between age groups (*p* = 0.95) (Figure S7). Similar results were found when studies were divided based on an older age (below or above 65 years and 70 years) (data not shown).

#### 3.5.3 Studies With > 50% of Female Subjects Versus Studies With < 50% of Female Subjects

We also performed analyses based on the prevalence of female subjects. Regarding the studies evaluating low muscle mass with adequate muscle strength and low muscle strength with adequate muscle mass, all studies included similar percentages of female subjects, all below 50% (from 33% to 47%). For confirmed sarcopenia, 14 papers had less than 50% [12, 15, 27, 31, 32, 34, 35, 36, 38, 39, 40, 41, 42, 43] and only two studies [28, 30] had more than 50% of female participants. One study [33] was not included because it did not report the prevalence of female subjects. Results showed an effect of sarcopenia in the studies including less than 50% females (HR < 50%: 2.10 [1.63; 2.72], *p* < 0.001) and a trend for the studies including more than 50% of females (HR > 50%: 2.93 [0.98; 8.73], *p* = 0.05), with no difference between subgroup homogeneity (*p* = 0.56) (Figure S8).

## 4 Discussion

In this systematic review and MA, we report that irrespective of the method applied, the cutoffs used, and the confounders considered, there was a consistent association between low muscle mass with adequate muscle strength, low muscle strength with adequate muscle mass, or the combination of low muscle mass and strength (sarcopenia) and increased risk of death in patients on dialysis. However, the magnitude of the association was strongest in the case of confirmed sarcopenia (2.02‐fold heightened risk), followed by low muscle strength with adequate muscle mass (82% increased risk)and low muscle mass with adequate muscle strength (49% increased risk). The heterogeneity was considered low for all analyses. Our data support previous meta‐analyses that evaluated the association between sarcopenia and mortality in dialysis patients [44], between HGS and mortality in patients on dialysis [45], and between sarcopenia and mortality in nondialysis patients [46]. Adding to the previous studies, we report the results of the diverse and isolated muscle abnormalities within the same study. Despite the low number of studies, our results show that low muscle strength had a higher weight on the outcome of death in comparison to low muscle mass, even when considering only the papers evaluating both muscle mass and muscle strength separately and combined. This finding is in agreement with the latest European consensus for diagnosing sarcopenia, where low muscle strength is used to define ‘probable sarcopenia’ [3, 4].

Our finding of the pooled MA where low muscle strength alone was associated with a higher risk for death highlights that assessing muscle strength can indeed be implemented in usual care, mainly because its assessment by handgrip dynamometer offers the advantage of being more easily assessed than muscle mass. The latter can be influenced by the altered hydration status, a condition that is often present in patients with CKD and kidney failure. However, our results are limited to the association with mortality and not with other clinical outcomes such as the increased occurrence of falls and fractures and the ability to perform activities of daily living. Finally, we acknowledge that differences in sex distribution and age among patients in the studies can influence the association between muscle abnormalities and the risk of death [47]. In our subgroup analysis by sex (> 50% female vs. < 50% female), which was only possible for the confirmed sarcopenia analysis, although presenting strong hazard ratios for both groups, in the group with a higher prevalence of female subjects the analysis was not significant (*p* = 0.05), and it probably depends on the very wide CIs of one of the studies included [30]. In our understanding, since low muscle mass and strength were identified with sex‐specific cutoffs in all the studies included, there should not be an important influence of sex on the risk of mortality, and the difference in the results found under this analysis should be considered a statistical artefact. On the other hand, our subgroup analysis by age (< 60 and ≥60 years) showed no difference in the association between muscle abnormalities and mortality, reinforcing the consistency of our findings.

Our findings agree with meta‐analyses in geriatric populations, suggesting that our conclusions may be valid and potentially generalisable to geriatric populations. For instance, a higher HGS was protective for mortality (HR: 0.72, 95% CI: 0.67 to 0.78) and for the incidence of disability (HR: 0.76, 95% CI: 0.66 to 0.87) [48]. Similarly, low muscle mass was associated with higher mortality risk [49]. Finally, for sarcopenia, an MA encompassing studies using different definitions of this diagnosis, a higher mortality risk was observed regardless of the definition applied [50].

The pathophysiological mechanisms of sarcopenia in older adults probably differ to some extent from that of patients on dialysis [1, 2] in that, among the latter, metabolic disturbances that increase protein degradation, inhibit protein synthesis, and increase energy expenditure are triggered both by uremic toxicity and the dialysis procedure. In addition, insufficient energy and nutrient intake, coupled with a sedentary lifestyle [51] may further exacerbate the catabolic processes. Like what is observed in patients with cancer, who are also exposed to catabolic conditions from the disease (effect of a malignant tumour) and from the treatment (chemotherapy and radiotherapy). One important characteristic of sarcopenia in kidney failure patients is that it can start early in adult life. In fact, the mean age of the patients included in our MA exceeded 50 years, while the mean age of participants in studies of sarcopenia in older adults without CKD/ESKD was > 70 years [48, 49, 50, 52]. In patients undergoing dialysis, many factors, such as inflammation, mitochondrial dysfunction, hyperparathyroidism, low appetite and effects linked to the dialysis procedure [53], that increase protein catabolism and can reduce food intake, likely reflect an overall worsening clinical condition that may explain the association of markers of poor muscle health with higher mortality risk.

Some strengths and limitations of our study deserve attention. We show that the association between sarcopenia traits and mortality differs between individuals with low muscle strength and adequate muscle mass and those with low muscle mass and adequate muscle strength; in addition to sarcopenia, his approach is the main novelty of this MA. We had a rigorous and extensive search of the literature following the PRISMA reporting guidelines [17] and GRADE system [23] to ensure the quality of the literature search, the degree of evidence, and add transparency to the results. We have attempted to minimize biases related to the initiation of the dialysis procedure by performing subgroup analyses for incident and prevalent patients. In addition, we improved rigour by selecting studies that diagnosed sarcopenia and its components only by applying objective measurements and not by questionnaires. Finally, because the studies included in the MA performed analyses of HR after adjusting for confounding variables, our results presented a strong effect with low heterogeneity (i.e., *I* ^2^ < 30%). However, some limitations need to be highlighted. First, the insufficient number of studies did not allow the analysis of other subgroups. Secondly, the lack of information about the level of physical activity in the original studies, as well as the presence of comorbidities such as diabetes and other chronic diseases, would not allow for sensitivity analysis. Finally, we recognize that studies showing associations with mortality are more likely to be published than studies showing a lack of association.

In conclusion, this systematic review and MA indicates that the association between low muscle mass with adequate muscle strength and mortality seems to be less strong than the association between low muscle strength with adequate muscle mass and mortality. The combination of both traits is strongly associated with the risk of dying for patients on dialysis. These results emphasize the value of routine assessment of these parameters, especially of low muscle strength by HGS in clinical practice. Ultimately, the question remains whether early identifying and treating sarcopenia or one muscle abnormality in these patients can be used to implement actions that could prevent poor outcomes. This needs to be addressed in the form of clinical trials.

## Conflicts of Interest

AS reports personal fees from Fresenius‐Kabi Deutschland GmbH and Baxter Healthcare Corporation during the conduct of the study but not related to the submitted work.

BL reports grants and personal fees from Baxter Healthcare Corporation during the conduct of the study, as well as grants and personal fees from Baxter Healthcare Corporation outside the submitted work.

PS reports personal fees from REATA and Baxter, grants and personal fees from Astra Zeneca, personal fees from BMS/Pfizer, Vifor, FMC and Astellas, outside the submitted work.

CMA reports personal fees from Baxter, Fresenius‐Kabi and AstraZeneca, all outside the submitted work.

None of the other authors have conflicts of interest to declare.

## Supporting information


**Supplementary Table 1:** Eligibility criteria for inclusion of studies in the meta‐analysis and systematic review.
**Supplementary Table 2:**_._ Detailed search strategy.
**Supplementary Table 3:** Summary of study characteristics and main results separated by measurement of interest (muscle mass, muscle strength and sarcopenia).
**Supplementary Table 4:** Summary of assessment of certainty of evidence (GRADE) for increased risk for mortality.
**Supplementary Figure 1:** Risk of bias.
**Supplementary Figure 2:** Galbraith plot of studies investigating the effect of confirmed sarcopenia included in the meta‐analysis.
**Supplementary Figure 3:** Funnel plot for the evaluation of publication bias for studies on sarcopenia.
**Supplementary Figure 4:** Subgroup analysis for the effect of low muscle mass with adequate muscle strength on mortality based on dialysis vintage (incident vs. prevalent patients).
**Supplementary Figure 5:** Subgroup analysis for the effect of low muscle strength with adequate muscle mass on mortality based on dialysis vintage (incident vs. prevalent patients).
**Supplementary Figure 6:** Subgroup analysis for the effect of confirmed sarcopenia on mortality based on dialysis vintage (incident vs. prevalent patients).
**Supplementary Figure 7:** Subgroup analysis for the effect of confirmed sarcopenia on mortality based on age (< 60 years vs. ≥ 60 years).
**Supplementary Figure 8:** Subgroup analysis for the effect of confirmed sarcopenia on mortality based on sex (> 50% females vs. < 50% females).

## Data Availability

The authors confirm that the data supporting the findings of this study is available within the article and its supplementary materials.
