# Supplementary material for: Sarcopenia and Its Individual Traits Independently Predict Mortality in Patients on Dialysis: A Systematic Review and Meta‐Analysis
Source: J Cachexia Sarcopenia Muscle. 2025 Oct 9;16(5):e70089. doi: 10.1002/jcsm.70089 (PMC12510902; doi:10.1002/jcsm.70089)

**Supplementary Material**

**Table of contents for the supplemental material**

| **Supplementary Table / Figure Number** | **Content / Title** |
| --- | --- |
| Supplementary Table 1 | Eligibility criteria for inclusion of studies in the meta-analysis and systematic review |
| Supplementary Table 2 | Detailed search strategy |
| Supplementary Table 3 | Summary of study characteristics and main results separated by measurement of interest (muscle mass, muscle strength and sarcopenia) |
| Supplementary Table 4 | Summary of assessment of certainty of evidence (GRADE) for increased risk for mortality |
| Supplementary Figure 1 | Risk of bias |
| Supplementary Figure 2 | Galbraith plot of studies investigating the effect of confirmed sarcopenia included in the meta-analysis |
| Supplementary Figure 3 | Funnel plot for the evaluation of publication bias for studies on sarcopenia |
| Supplementary Figure 4 | Subgroup analysis for the effect of low muscle mass with adequate muscle strength on mortality based on dialysis vintage (incident versus prevalent patients). |
| Supplementary Figure 5 | Subgroup analysis for the effect of low muscle strength with adequate muscle mass on mortality based on dialysis vintage (incident versus prevalent patients). |
| Supplementary Figure 6 | Subgroup analysis for the effect of confirmed sarcopenia on mortality based on dialysis vintage (incident versus prevalent patients). |
| Supplementary Figure 7 | Subgroup analysis for the effect of confirmed sarcopenia on mortality based on age (<60 years versus ≥ 60 years). |
| Supplementary Figure 8 | Subgroup analysis for the effect of confirmed sarcopenia on mortality based on sex (>50% females versus < 50% females). |

**Supplementary Table 1:** Eligibility criteria for inclusion of studies in the meta-analysis and systematic review.

| **Parameter** | **Inclusion Criteria** |
| --- | --- |
| Population | Patients over 18 years old on dialysis treatment (hemodialysis and peritoneal dialysis) |
| Exposure | Low muscle mass and/or low muscle strength (muscle strength combined for sarcopenia diagnosis or assessed as isolated conditions |
| Comparison | Groups comprised by patients with adequate muscle mass and/or muscle strength |
| Outcome | Survival follow-up for at least a year |
| Study design | Retrospective or prospective observational and longitudinal studies |

**Supplementary Table 2.** Detailed search strategy

| Free text words and MeSH terms applied in the search strategy |
| --- |
| ("Chronic kidney disease" OR Hemodialysis OR "Peritoneal dialysis" OR Dialysis OR "End-stage kidney disease" OR "End-stage renal disease" OR "Chronic kidney failure" OR "Chronic renal disease" OR "Chronic renal insufficiency" OR "Chronic Kidney Insufficiency" OR CKD OR ESKD OR ESRD) AND (sarcopenia OR "muscle mass" OR "lean body mass index" OR "lean body mass" OR "fat body mass index" OR "fat body mass" OR "lean mass index" OR "lean mass" OR "appendicular skeletal muscle index" OR "appendicular skeletal muscle mass" OR "muscle strength" OR "hand grip strength" OR dynamometer OR "handgrip dynamometer" OR "isokinetic dynamometer" OR "muscle functionality" OR "muscle function" OR "physical performance" OR "gait speed" OR "timed up and go test" OR "short-physical performance battery" OR "stair climb power test" OR " 6-min walk test" OR " 2-min step test" OR "Fat free mass" OR "physical function") AND (mortality OR "mortality risk" OR survival OR lethality OR fatality OR mortal OR death OR fatal OR "death rate" OR fateful OR "mortality rate"). |
| Pubmed |
| (("Chronic kidney disease"[All Fields] OR ("haemodialysis"[All Fields] OR "renal dialysis"[MeSH Terms] OR ("renal"[All Fields] AND "dialysis"[All Fields]) OR "renal dialysis"[All Fields] OR "hemodialysis"[All Fields]) OR "Peritoneal dialysis"[All Fields] OR ("dialysance"[All Fields] OR "dialysances"[All Fields] OR "dialysation"[All Fields] OR "dialysator"[All Fields] OR "dialysators"[All Fields] OR "dialyse"[All Fields] OR "dialysed"[All Fields] OR "dialyser"[All Fields] OR "dialysers"[All Fields] OR "dialysing"[All Fields] OR "dialysis solutions"[Pharmacological Action] OR "dialysis solutions"[MeSH Terms] OR ("dialysis"[All Fields] AND "solutions"[All Fields]) OR "dialysis solutions"[All Fields] OR "dialysate"[All Fields] OR "dialysates"[All Fields] OR "dialyzate"[All Fields] OR "dialyzates"[All Fields] OR "dialysis"[MeSH Terms] OR "dialysis"[All Fields] OR "dialyses"[All Fields] OR "dialyzability"[All Fields] OR "dialyzable"[All Fields] OR "dialyzation"[All Fields] OR "dialyze"[All Fields] OR "dialyzed"[All Fields] OR "dialyzer"[All Fields] OR "dialyzer s"[All Fields] OR "dialyzers"[All Fields] OR "dialyzing"[All Fields] OR "renal dialysis"[MeSH Terms] OR ("renal"[All Fields] AND "dialysis"[All Fields]) OR "renal dialysis"[All Fields]) OR "End-stage kidney disease"[All Fields] OR "End-stage renal disease"[All Fields] OR "Chronic kidney failure"[All Fields] OR "Chronic renal disease"[All Fields] OR "Chronic renal insufficiency"[All Fields] OR "Chronic Kidney Insufficiency"[All Fields] OR "CKD"[All Fields] OR "ESKD"[All Fields] OR ("kidney failure, chronic"[MeSH Terms] OR ("kidney"[All Fields] AND "failure"[All Fields] AND "chronic"[All Fields]) OR "Chronic kidney failure"[All Fields] OR "esrd"[All Fields])) AND ("sarcopenia"[MeSH Terms] OR "sarcopenia"[All Fields] OR "muscle mass"[All Fields] OR "lean body mass index"[All Fields] OR "lean body mass"[All Fields] OR "fat body mass index"[All Fields] OR "fat body mass"[All Fields] OR "lean mass index"[All Fields] OR "lean mass"[All Fields] OR "appendicular skeletal muscle index"[All Fields] OR "appendicular skeletal muscle mass"[All Fields] OR "muscle strength"[All Fields] OR "hand grip strength"[All Fields] OR ("dynamometer"[All Fields] OR "dynamometers"[All Fields]) OR "handgrip dynamometer"[All Fields] OR "isokinetic dynamometer"[All Fields] OR "muscle functionality"[All Fields] OR "muscle function"[All Fields] OR "physical performance"[All Fields] OR "gait speed"[All Fields] OR "timed up and go test"[All Fields] OR "short-physical performance battery"[All Fields] OR "stair climb power test"[All Fields] OR "6-min walk test"[All Fields] OR "2-min step test"[All Fields] OR "Fat free mass"[All Fields] OR "physical function"[All Fields]) AND ("mortality"[MeSH Terms] OR "mortality"[All Fields] OR "mortalities"[All Fields] OR "mortality"[MeSH Subheading] OR "mortality risk"[All Fields] OR ("mortality"[MeSH Subheading] OR "mortality"[All Fields] OR "survival"[All Fields] OR "survival"[MeSH Terms] OR "survivability"[All Fields] OR "survivable"[All Fields] OR "survivals"[All Fields] OR "survive"[All Fields] OR "survived"[All Fields] OR "survives"[All Fields] OR "surviving"[All Fields]) OR ("lethal"[All Fields] OR "lethalities"[All Fields] OR "lethality"[All Fields] OR "lethals"[All Fields]) OR ("fatal"[All Fields] OR "fatalities"[All Fields] OR "fatality"[All Fields] OR "fatally"[All Fields]) OR ("mortal"[All Fields] OR "mortally"[All Fields] OR "mortals"[All Fields]) OR ("death"[MeSH Terms] OR "death"[All Fields] OR "deaths"[All Fields]) OR ("fatal"[All Fields] OR "fatalities"[All Fields] OR "fatality"[All Fields] OR "fatally"[All Fields]) OR "death rate"[All Fields] OR "fateful"[All Fields] OR "mortality rate"[All Fields])) AND (2021/1/5:2021/10/20[pdat]) |
| Embase |
| ('chronic kidney disease' OR hemodialysis OR 'peritoneal dialysis' OR dialysis OR 'end-stage kidney disease' OR 'end-stage renal disease' OR 'chronic kidney failure' OR 'chronic renal disease' OR 'chronic renal insufficiency' OR 'chronic kidney insufficiency' OR ckd OR eskd OR esrd) AND (sarcopenia OR 'muscle mass' OR 'lean body mass index' OR 'lean body mass' OR 'fat body mass index' OR 'fat body mass' OR 'lean mass index' OR 'lean mass' OR 'appendicular skeletal muscle index' OR 'appendicular skeletal muscle mass' OR 'muscle strength' OR 'hand grip strength' OR dynamometer OR 'handgrip dynamometer' OR 'isokinetic dynamometer' OR 'muscle functionality' OR 'muscle function' OR 'physical performance' OR 'gait speed' OR 'timed up and go test' OR 'short-physical performance battery' OR 'stair climb power test' OR '6-min walk test' OR '2-min step test' OR 'fat free mass' OR 'physical function') AND (mortality OR 'mortality risk' OR survival OR lethality OR fatality OR mortal OR death OR fatal OR 'death rate' OR fateful OR 'mortality rate') AND [5-1-2021]/sd NOT [21-10-2021]/sd |
| Web of Science |
| ('chronic kidney disease' OR hemodialysis OR 'peritoneal dialysis' OR dialysis OR 'end-stage kidney disease' OR 'end-stage renal disease' OR 'chronic kidney failure' OR 'chronic renal disease' OR 'chronic renal insufficiency' OR 'chronic kidney insufficiency' OR ckd OR eskd OR esrd) AND (sarcopenia OR 'muscle mass' OR 'lean body mass index' OR 'lean body mass' OR 'fat body mass index' OR 'fat body mass' OR 'lean mass index' OR 'lean mass' OR 'appendicular skeletal muscle index' OR 'appendicular skeletal muscle mass' OR 'muscle strength' OR 'hand grip strength' OR dynamometer OR 'handgrip dynamometer' OR 'isokinetic dynamometer' OR 'muscle functionality' OR 'muscle function' OR 'physical performance' OR 'gait speed' OR 'timed up and go test' OR 'short-physical performance battery' OR 'stair climb power test' OR '6-min walk test' OR '2-min step test' OR 'fat free mass' OR 'physical function') AND (mortality OR 'mortality risk' OR survival OR lethality OR fatality OR mortal OR death OR fatal OR 'death rate' OR fateful OR 'mortality rate') AND [5-1-2021]/sd NOT [21-10-2021]/sd |
| Proquest |
| ("Chronic kidney disease" OR Hemodialysis OR "Peritoneal dialysis" OR Dialysis OR "End-stage kidney disease" OR "End-stage renal disease" OR "Chronic kidney failure" OR "Chronic renal disease" OR "Chronic renal insufficiency" OR "Chronic Kidney Insufficiency" OR CKD OR ESKD OR ESRD) AND (sarcopenia OR "muscle mass" OR "lean body mass index" OR "lean body mass" OR "fat body mass index" OR "fat body mass" OR "lean mass index" OR "lean mass" OR "appendicular skeletal muscle index" OR "appendicular skeletal muscle mass" OR "muscle strength" OR "hand grip strength" OR dynamometer OR "handgrip dynamometer" OR "isokinetic dynamometer" OR "muscle functionality" OR "muscle function" OR "physical performance" OR "gait speed" OR "timed up and go test" OR "short-physical performance battery" OR "stair climb power test" OR " 6-min walk test" OR " 2-min step test" OR "Fat free mass" OR "physical function") AND (mortality OR "mortality risk" OR survival OR lethality OR fatality OR mortal OR death OR fatal OR "death rate" OR fateful OR "mortality rate") |
| Google |
| With all of the words: “Chronic kidney disease”  With at least one of the words: sarcopenia OR "muscle mass" OR "lean body mass index" OR "lean body mass" OR "fat body mass index" OR "fat body mass" OR "lean mass index" OR "lean mass" OR "appendicular skeletal muscle mass"  Where my words occurs: anywhere in the article  200 most relevant hits |

**Supplementary Table 3.** Summary of study characteristics and main results separated by measurement of interest.

1. Muscle mass

| **Author, year** | **Country** | **HD/PD** | **Follow-up** | **Sample size (% female)** | **Age**  **(year)** | **Method to evaluate muscle mass and cutoff to classify low muscle mass** | **Muscle mass (baseline)** | **Low-muscle mass (%)** | **Mortality (%)** | **Mortality risk analysis** | **Adjusted variables in analysis** |
| --- | --- | --- | --- | --- | --- | --- | --- | --- | --- | --- | --- |
| Giglio J et al. 2018 (1) | Brazil | HD | Median 17.5 (IQR 12-31) months | 170 (34.7%) | 70.6 ± 7.2 | Feature: ASMI (Kg/m^2^)  Method: DXA  Cut off: ASMI/ height^2^  Men < 7.26 Kg/m^2^  Women < 5.45 Kg/m^2^ | Not available | Low ASMI in general: 109/170  (63.4%)  Low ASMI alone: 47/170 (27.6%) | 48/170 (28.2%) | Low ASMI alone: Unadjusted HR 1.05 (0.36-3.04), P = 0.924  Adjusted HR 1.03 (0.36-2.99), P = 0.953. | Age, gender, dialysis vintage, and DM |
| Yamamoto S et al. 2020 (2) | Japan | HD | Median 36 (IQR: 18 – 68.4) months | 542 (40%) | 65.3 ± 12.1 | Feature: Modified Creatinine Index (MCI) (mg/kg/day)  Cut off:  Median value (MCI <20.8 mg/kg/day) | MCI: 20.9 ±2.7 mg/kg/day | Low MCI in general: 271/542 (50%)  No information on the prevalence of Low MCI alone. | 138/542  (25.4%) | Low MCI normal strength  Adjusted model  HR: 2.14 (1.10-4.16); P<0.05 | Age, gender, BMI, dialysis vintage, comorbidity index, and serum albumin |
| Isoyama N et al. 2014 (3) | Sweden | HD  (incident patients) | 29 (1 – 48) months | 330 (38%) | 53±13 | Feature: ASMI (kg/m^2^)  Method: BIA  Cut off:  Men:<7.3 kg/m^2^  Women: <5.5 kg/m^2^ | Not available | Total prevalence:  Low muscle mass alone: 79/330 (24%) | 95/330  (29%) | Unadjusted  Low ASMI alone  HR: 1.35 (0.67-2.68), P = 0.39  Adjusted  Low ASMI alone  HR: 1.23 (0.56-2.67), P = 0.59 | Age, sex diabetes, CVD, cholesterol, hemoglobin, GFR, and hsCRP. |
| Xu X et al. 2020 (4) | China | PD  (incident patients) | 35 (16 – 72) months | 327 (47.6%) | 56.1 ± 15 | Feature: LBMI Method: equation  LBM (kg) = (1 if male; 0 if female) x 4.72 + height  (cm) x 0.28 + weight (kg) x 0.27 + HGS (N) x 0.02—  dialysis duration (months) x 0.04—26.84  LBMI = LBM/Height^2^  Cut-off LBMI:  Men: <16.7 Kg/m^2^  Women: <13.8 Kg/m^2^ | 15.6 ± 2.0 | Low muscle mass alone: 132/327 (40.4%) | 144/327 (44%) | Unadjusted  Low LMI HR: 1.24 (0.66-2.34), P = 0.501  Adjusted  Low LMI HR: 1.49 (0.79-2.82), P = 0.217 | Age, diabetes, cardiovascular disease and serum albumin |
| Kim JK et al. 2019 (5) | South Korea | HD | 4.3 ± 0.8 years | 142 (33%) | 59.8±13.1 | Feature: LTI by BIS  Cut-off: ≥ 2SDs below the normal gender-specific means for young people. | By sarcopenic status  Sarcopenic: 11.0±2.2  Non-sarcopenic: 13.9±2.9 | 14.1% low muscle mass and appropriate muscle strength | 28/142 (19.7%) | Log-rank = 0.011 |  |
| Sabatino et al. 2024 (6) | Italy | HD | 28 (IQR 19-36) | 99 (30%) | 66±17 | Feature: Distal QVI thickness  Method: US  Cut-off:  Men <3.44 mm/m^2^  Women <3.52 mm/m^2^ | 3.6 ± 1.3 mm/m^2^ | Low QVI thickness index in general: 52/99 (52%)  Low QVI thickness index alone: 8/64 (12.5%) | 38/99 (38.4%)  No subjects with only low muscle mass died | No subjects with only low muscle mass died |  |

ASMI: Appendicular skeletal muscle index; BIA: Bioelectrical impedance analysis; BIS: Bioimpendance spectroscopy; BMI: Body mass index; DM: Diabetes mellitus; DXA, Dual energy X-ray absorptiometry; HD: Hemodialysis; HGS: Handgrip strength; HR, Hazard-ratio; hsCRP, high sensitive C-reactive protein; LBM: Lean body mass; LBMI: Lean body mass index; LTI: Lean tissue index; MCI: Modified creatinine index; PD: peritoneal dialysis; QVI, Quadriceps vastus intermedius.

1. Muscle strength.

| **Author, year** | **Country** | **HD/PD** | **Follow-up** | **Sample size (percent female)** | **Age (years)** | **Muscle strength assessment method and cut-off** | **Muscle strength (baseline)** | **Low muscle strength (%)** | **Mortality (%)** | **Mortality risk analysis** | **Adjusted variables in analysis** |
| --- | --- | --- | --- | --- | --- | --- | --- | --- | --- | --- | --- |
| Giglio J et al. 2018 (1) | Brazil | HD | Median 17.5 (IQR 12-31) months | 170 (34,7%) | 70.6 ± 7.2 | Feature: HGS (Kg)  Cut off:  Men < 30 kg;  Women < 20 kg | Not available | Low HGS in general: 88/170 (51.7%)  Low HGS alone: 47/170 (27.6%) | 48/170 (28.2%) | Low HGS alone  Non-adjusted HR: 1.50 (0.51-4.47), P = 0.465  Adjusted HR:1.43 (0.46-4.39), P = 0.533 | Age,  gender, dialysis vintage, and DM |
| Souweine JS et al. 2020 (7) | France | HD | Mean 23.7 (range 16.8-34.9) months | 187  (35%) | Median 65.3 (49.7-82) | Feature: Maximal Voluntary Force (MVF) of quadriceps  Cut off: below the median  Dynapenia: low MVF and normal lean body mass | MVF: 88.1 (48.5-127.8) N.m. | Low MVF alone (dynapenia): 30/187  (16%) | 44/187 (23.5%) | Patients with dynapenia  Unadjusted HR: 2.2 (0.9-5.4); P = 0.084  Adjusted HR: 2.99 (1.18-7.61); P = 0.02 | Age, gender, LTI,  serum albumin, hs-CRP, haemoglobin, nPCR, dialysis vintage and Charlson score |
| Yamamoto S et al. 2020 (2) | Japan | HD | Median 36 (IQR: 18 – 8.4) months | 542 (40%) | 65.3 ± 12.1 | Feature: HGS (Kg);  Cut off:  Men <28 kg  Women < 18 kg | 24.7 ± 8.7 kg | Not described | 138/542 (25.4%) | Low HGS alone  Adjusted HR:  1.85 (1.07-3.21), P < 0.05 | Age, gender, BMI, dialysis length, comorbidity index, and serum albumin |
| Sabatino et al. 2024 (6) | Italy | HD | 28 (IQR 19-36) | 99 (30%) | 66±17 | Feature: HGS  Cut-off:  Men <27Kg  Women <16Kg | 22.4 ± 8.6 | Low HGS in general: 39/64 (61%)  Low HGS alone: 16/64 (25%) | 38/99 (38.4%) | Low HGS alone  Adjusted: 2.02 (0.38-10.69), P = 0.407 | Serum albumin and age-corrected Charlson comorbidity index |
| Elder et al, 2023 (8) | Australia | HD | 72 months | 77 (42.8%) | Median  71 (60-87) | Feature: HGS  Cut-off:  Men <27Kg  Women <16Kg | 22.9 ± 8.7 | Low HGS alone: 10/77 (13%) | 50/77 (65%) | Low HGS alone  Unadjusted HR: 1.24 (0.52-2.97), P = 0.62  Adjusted HR: 0.79 (0.31-2.01), P = 0.760 | Dialysis vintage, age, comorbidity score, MAP |
| Xu et al, 2020 (4) | China | PD (incident patients) | 35 (IQR 16-72) | 327 (47.6%) | 56.1 ± 15.0 | Feature: HGS  Cut-off:  Men <24.5 Kg  Women <14 Kg | 21.8 ± 10.7 | Low HGS alone: 47/327 (14.4%) | 144/327 (44%) | Low HGS alone  Unadjusted HR: 3.0 (1.87-4.80), P < 0.001  Adjusted HR: 1.72 (1.05-2.80), P = 0.030 | Age, DM, CVD and serum albumin |
| Isoyama et al, 2014 (3) | Sweden | HD  (incident patients) | 29 (1 – 48) months | 330 (38%) | 53±13 | Feature: HGS  Cut-off  Men <30Kg  Women <20Kg | Not mentioned | Low HGS alone: 50/330 (15.1%) | 95/330 (28.8%) | Low HGS alone  Unadjusted HR: 2.82 (1.57-5.21), P = 0.001  Adjusted HR: 1.98 (1.01-3.87), P = 0.04 | Age, sex, DM, CVD, cholesterol, hemoglobin, GFR, hsCRP. |

CRP: C reactive protein; DM: Diabetes mellitus; HD:Hemodialysis; GFR: Glomerular filtration rate; HGS: Handgrip strength; hs-CRP: high-sensitivity C-reactive protein; MAP, mean arterial pressure; MIS: malnutrition inflammation score; MNA-SF: Mini-nutritional assessment – Short form; MVF: Maximal volountary force; ; nPCR: normalized Protein catabolic rate; PD: Peritoneal dialisys;

C. Sarcopenia

| **Author, year** | **Country** | **HD/PD** | **Follow-up** | **Sample size (percent female)** | **Age (years)** | **Sarcopenia diagnosis** | **Sarcopenia (%)** | **% Mortality** | **Mortality risk analysis** | **Adjusted variables in analysis** |
| --- | --- | --- | --- | --- | --- | --- | --- | --- | --- | --- |
| Kittiskulnam et al, 2017 (9) | US | HD | 22.8 (range 1-38.4) months | 645 (41.4%) | 56.7 ± 14.5 | Low SMI by BIS and low HGS  Low SMI: 2 SD or more below sex specific of healthy adults.  Low muscle strength: Men <26 kg; women <16 kg. | 25/643 (3.9%) | 78/645 (12.1%) | Sarcopenia Unadjusted HR: 4.23 (2.11-8.49), P = 0.001  Adjusted HR: 2.23 (0.99-5.90), P = 0.05 | Age, sex, race, comorbidities (DM, CHF, CAD), serum albumin |
| Mori K et al, 2019 (10) | Japan | HD | Median 90  (IQR 44–108) months | 308 (40%) | Non-sarcopenic 54.4 ± 11.0  Sarcopenic 63.5 ± 11.0 | Low SMI by DXA and low HGS  Low SMI: Men <7 kg/m^2^; women <5.4 kg/m^2^  Low muscle strength: Men <26 kg; women <18 kg. | 144/308 (40%) | 100/308  (32.5%) | Sarcopenia Adjusted HR: 1.31 (0.81-2.10); P = 0.268 | Age, HD vintage, Gender, BMI, DM, serum albumin, Kt/V, nPCR |
| Giglio J et al, 2018 (1) | Brazil | HD | Median 17.5 (IQR 12-31) months | 170 (34.7%) | 70.6 ± 7.2 | Low ASMI by DXA and low HGS  Cut off: ASMI/ height^2^  M < 7.26 Kg/m^2^  F < 5.45 Kg/m^2^  Low muscle strength: Men <30 kg; women <20kg. | 62/170 (36.4%) | 48/170  (28.2%) | Sarcopenia: Unadjusted HR 2.02 (1.14-3.57), P = 0.016.  Adjusted HR 2.09 (1.05-4.20), P = 0.037 | Age, gender, dialysis vintage, and DM |
| Kim JK et al, 2019 (5) | South Korea | HD | 51.6 ± 9.6 months | 142 (47.2%) | 59.8 ± 13.1 years (range, 21 to 88) | Low LTI by BIS and low HGS  Low LTI: ≥ 2SDs below the normal gender-specific means for young people  Low muscle strength: Men <30 kg; women <20kg. | 47/142 (33.1%) | 28/142  (19.7%) | Sarcopenia Adjusted HR: 6.99 (1.84-26.58); P = 0.004 | Age, gender, BMI, Kt/V, albumin, DM, dialysis vin­tage, hs-CRP, CAD, and cerebrovascular disease |
| Souweine JS et al, 2020 (7) | France | HD | 23.7 (12.4-34.9) months | 187 (35%) | 65.3 (49.7-82.0) | Low muscle mass (CI) and low MVF of the quadriceps.  Muscle strength and muscle mass below of MVF and CI (no specific cutoffs were given). | 63/187 (33.7%) | 44/187 (23.5%) | Sarcopenia Unadjusted HR: 3 (1.5-6.0); P = 0.002  Adjusted HR: 1.6 (0.76-3.35); P = 0.21 |  |
| YL et al, 2020 (11) | Taiwan | HD | 36 months | 126 (48.4%) | 63.2 ± 13 | Low SMI by BIA and low HGS by two criteria: European and Taiwanese  Cut off: ASMI/ height^2^  Men < 8.87 Kg/m^2^  Women < 6.42 Kg/m^2^  Low muscle strength: Men <26 kg; women <18kg. | European criteria 11/126 (8.7%) Taiwanese criteria: 17/126 (13.5%) | 26/126  (20.6%) | Sarcopenia Kaplan-Meier curve  Log-rang, P = 0.037 | NA |
| Yamamoto S et al, 2020 (2) | Japan | HD | 36 (IQR: 18 – 68.4) months | 542 (40%) | 65.3 ± 12.1 | Low modified creatinine index and low HGS  Low modified creatinine index: <20.8 mg/kg/day  Low HGS: Men <28 kg; women < 18 kg | Not described | 138/542 (25.4%) | Sarcopenia Adjusted HR: 3.79 (2.09-6.87); P<0.001 | Age, gender, BMI, dialysis length, comorbidity index, and serum albumin |
| Song YR et al, 2020 (12) | Korea | HD | 62.4 months | 88 (43.2%) | 60.6 ± 13.5 | Low LTI by BIS and low HGS  Low LTI:  <10^th^ percentile for reference population (no cutoffs given).  Low HGS: Men <30 Kg; women <20 Kg | 36/88 (40.9%) | 30/88 (34.1%) | Sarcopenia Unadjusted HR: 7.71 (1.83-32.57); P = 0.018  Adjusted HR: 2.72; 95% CI (1.11–6.63); P = 0.028 | Age, gender, BMI, dialysis length, comorbidity index, and serum albumin |
| Elder et al, 2023 (8) | Australia | HD | 72 months Patients who died: 33.6 (range 14.4-56.4) months | 77 (42.8%) | 71 (range 60-87) | Low LTI by BIS and low HGS  < 2SD from healthy gender-specific reference values (no cutoffs given).  Low HGS: Men <27Kg; women <16Kg | 33/77 (42.9%) | 50/77 (65%) | Sarcopenia Unadjusted HR: 0.96 (0.49-1.91); P = 0.91  Adjusted HR: 0.89 (0.44; 1.84); P = 0.76 | Age, dialysis vintage, comorbidity score, MAP |
| Correa et al, 2023 (13) | Brazil | HD | 60 months | 247 (39.3%) | 66.6 ± 3.5 | Low FFMI by DXA and low HGS  Low FFMI: Men < 15.3 kg/m^2^; women <15.6 kg/m^2^ | 54/247 (21.9%) | 38/247 (15.4%) | Sarcopenia Adjusted HR: 3.34 (1.61-6.91); P = 0.001 | Age, sex, obesity, use of statins |
| Ishimura et al, 2022 (14) | Japan | HD | 76 ± 35 months | 308 (40%) | 58.0 ± 11.9 | Low SMI by DXA and low HGS  Low SMI: Men <7.0 kg/m^2^; women <5.4 kg/m^2^  Low muscle strength: Men <28kg; women <18kg. | Sarcopenia: 83/308 (26.9%) Sarcopenic obesity: 48/308 (15.6%) | 100/308 (32.5%) | Sarcopenia Adjusted HR: 1.15 (0.75-1.77); P = 0.528  Sarcopenic obesity HR: 1.275 (0.78-2.09); P = 0.326 | Age, sex, DM |
| Sanchez-Tocina et al, 2022 (15) | Spain | HD | 24 months | 60 (32%) | 81.9 ± 5.6 | Low ASM by BIA and low HGS  Cutoffs for low ASM and HGS: not described. | In general 24/60 (40%) Sarcopenia by low HGS: 23/60 (38%) Sarcopenia by low STS: 22/60 (37%) | 30/60 (50%) | Sarcopenia Unadjusted HR: 2.5 (1.13-5.55); P = 0.024  Adjusted HR: 2.04 (0.89-4.71); P = 0.094 | Age, cardiovascular disease |
| Zhang et al, 2022 (16) | China | HD | 18 months | 158 (50.6%) | 54 ± 14 | Low SMI by BIA and low HGS or low GS (Asian working Group Criteria)  Low SMI: Men <7.0 kg/m^2^; women <5.7 kg/m^2^  Low muscle strength: Men <28kg; women <18kg.  Low GS: < 1m/s | 46/158 (29.1%) | 11/158 (6.9%) | Sarcopenia Adjusted HR: 6.59 (1.08-39.91); P = 0.041 | Age, CVD, serum albumin, BMI, Kt/V, DM, hypertension, CRP, low SMI, HGS, LVMI |
| Isoyama et al, 2014 (3) | Sweden | HD  (incident patients) | 29 (1 – 48) months | 330 (38%) | 53±13 | Low ASMI by DXA and low HGS  Low SMI: Men <7.3 kg/m^2^; women <5.5 kg/m^2^  Low muscle strength: Men <30 kg; women <20kg | 68/330 (20.6%) | 95/330 (28.8%) | Sarcopenia Unadjusted HR: 2.94 (1.64-5.27); P < 0.001  Adjusted HR: 1.93 (1.01-3.71); P = 0.04 | Age, sex, DM, CVD, cholesterol, hemoglobin, GFR, hsCRP. |
| Xu et al, 2020 (4) | China | PD (incident patients) | 35 (IQR 16-72) | 327 (47.6%) | 56.1 ± 15.0 | Low LMI calculated from equation: LBM (kg) = (1 if male; 0 if female) x 4.72 + height  (cm) x 0.28 + weight (kg) x 0.27 + HGS (N) x 0.02—  dialysis duration (months) x 0.04—26.84  LBMI = LBM/Height^2^  and low HGS  Low LMI:  Men: <16.7 Kg/m^2^  Women: <13.8 Kg/m^2^  Low HGS: Men <24.5 Kg; women <14 Kg | 86/327 (26.3%) | 144/327 (44%) | Sarcopenia Unadjusted HR: 3.7 (2.45-5.59); P < 0.001  Adjusted HR: 2.49 (1.61-3.85); P < 0.001 | Age, DM, CVD and serum albumin |
| Fantinel Ferreira et al, 2022 (17) | Brazil | HD | 23.5 (IQR 14.9-29) months | 127 (39.4%) | 40% > 60 years old | Low Calf circumference and low HGS  Low calf circumference: Men <34 cm; women <33 cm  Low HGS: Men <30 Kg; women <20 Kg | 34/127 (26.8%) | 36/127 (28.3%) | Sarcopenia Unadjusted HR: 3.95 (2.05-7.61); P < 0.001  Adjusted HR: 2.98 (1.44-6.13); P = 0.003 | Age, DM, COPD, CHF, HIV, Hepatitis C |
| Xiang et al, 2023 (18) | China | HD | 35.1 ± 15.4 | 209 (52.6%) | 58.4 ± 15.3 | Low ASMI by BIA and low HGS (Asian working group for Sarcopenia criteria)  Low ASMI: Men <7.0 kg/m^2^; women <5.4 kg/m^2^  Low HGS: Men <26 Kg; women <18 Kg | 78/209 (37.3%) | 99/209 (47.6%) | Sarcopenia Unadjusted HR: 3.15 (1.13-8.7); P = 0.028  Adjusted HR: 0.36 (0.53-5.95); P = 0.356  Osteosarcopenia HR: 3.74 (1.17-11.9); P = 0.026 | Age, sex, dialysis vintage, DM, CVD, fracture history |
| Sabatino et al, 2024 (6) | Italy | HD | 28 (IQR 19-36) | 99 (30%) | 66±17 | Low VI thickness by US and low HGS  Low VI Thicknesses: Men <3.44 mm/m^2^  Women <3.52 mm/m^2^  Low HGS: Men <27 Kg; Women <16 Kg | 23/78 (30%) | 38/99 (38.4%) | Sarcopenia Unadjusted HR: 4.46 (2.08-9.56); P < 0.001  Adjusted HR: 3.21 (1.37-7.53); P = 0.007 | Serum albumin and age-corrected Charlson comorbidity index |
| Ren H et al, 2016 (19) | China | HD | 12 months | 131 (38.9%) | 49.4 ± 11.7 (23–72) | Low SMMI by MF-BIA and low HGS  Low SMMI: Men <8.51 kg/m^2^; women < 5.76 kg/m^2^  Low HGS: Men <30 Kg; Women <20 Kg | 18/131 (13.7%) | 3/131 (2.2%) | Sarcopenic group: 88.9% one-year survival rate, lower than the non-sarcopenic group (P = 0.007) | NA |

ASMI: appendicular smooth muscle index; BIA: Bioimpendace Absorptiometry; BIS: Bioimpendance spectroscopy; CAD: Chronic artery disease; CHF: Chronic heart failure; CI: Creatinine index; COPD: Chronic obstructive pulmonary disease; CVD: Cardiovascular disease; DM: Diabetes mellitus; DXA: Dual x-ray Absorptiometry; FFMI: free fat mass index; HD: Hemodialysis; HGS: Handgrip Strength; hs-CRP: high-sensitivity C-reactive protein; LTI: Lean tissue index; MCI: Modified creatinine index; LVMI, Left ventricular mass index; MCI: Modified creatinine index; MF-BIA: Multifrequency bioelectrical impendance Analysis; MVF: Maximum voluntary force; nPCR: normalized Protein catabolic rate; SMI: Skeletal muscle index ; SMMI: Skeletal muscle mass index; US: Ultrasound; VI: Vastus intermedius; SD: Standard deviation

**Supplementary Table 4.** Summary of assessment of certainty of evidence (GRADE) (1) for increased risk for mortality

|  | **Certainty assessment** | | | | | | | **Effect** | **Certainty of evidence** |
| --- | --- | --- | --- | --- | --- | --- | --- | --- | --- |
| **Type of exposition** | **№ of studies** | **Study design** | **Risk of bias** | **Inconsistency** | **Indirectness** | **Imprecision** | **Other considerations** | **Relative (95% CI)** |  |
| Low muscle mass | 4 | Observational studies | Not serious | Not serious | Not serious | Not serious | Publication bias strongly suspected^a^ | **HR 1.49** (1.04 to 2.13) | ⨁◯◯◯ Very low |
| Low muscle strength | 7 | Observational studies | Not serious | Not serious | Not serious | Not serious | Publication bias strongly suspected^a^ | **HR 1.82** (1.38 to 2.41) | ⨁◯◯◯ Very low |
| Confirmed sarcopenia | 17 | Observational studies | Not serious | Not serious | Not serious | Not serious | Strong association | **HR 2.02** (1.61 to 2.54) | ⨁⨁⨁◯ Moderate |

^a^The number of studies available was very low and didn't allow for publication bias evaluation. **CI:** confidence interval; **HR:** hazard Ratio.

1. Guyatt GH, Oxman AD, Schünemann HJ, Tugwell P, Knottnerus A. GRADE guidelines: a new series of articles in the Journal of Clinical Epidemiology. J Clin Epidemiol. 2011;64(4):380-2.

**Supplementary Figure 1.** Risk of bias

|  | Q1 | Q2 | Q3 | Q4 | Q5 | Q6 | Q7 | Q8 | Q9 | Q10 | Q11 |
| --- | --- | --- | --- | --- | --- | --- | --- | --- | --- | --- | --- |
| Kittiskulnam et al, 2017 (9) | 1 | 1 | 1 | 1 | 1 | 1 | 1 | 1 | 2 | 1 | 1 |
| Mori K et al, 2019 (10) | 1 | 1 | 1 | 1 | 1 | 1 | 1 | 1 | 1 | 1 | 1 |
| Giglio J et al, 2018 (1) | 1 | 1 | 1 | 1 | 1 | 1 | 1 | 1 | 1 | 1 | 1 |
| Kim JK et al, 2019 (5) | 1 | 1 | 1 | 1 | 1 | 1 | 1 | 1 | 3 | 1 | 1 |
| Ren H et al, 2016 (19) | 1 | 1 | 2 | 2 | 4 | 1 | 1 | 1 | 2 | 1 | 2 |
| Souweine JS et al, 2020 (7) | 1 | 1 | 1 | 1 | 1 | 1 | 1 | 1 | 1 | 1 | 1 |
| Lin YL et al, 2020 (11) | 1 | 1 | 1 | 2 | 2 | 1 | 1 | 1 | 1 | 1 | 2 |
| Yamamoto S et al, 2020 (2) | 1 | 1 | 1 | 1 | 1 | 1 | 1 | 1 | 1 | 1 | 1 |
| Song YR et al, 2020 (12) | 1 | 1 | 1 | 1 | 1 | 1 | 1 | 1 | 1 | 1 | 1 |
| Elder et al, 2023 (8) | 1 | 1 | 1 | 1 | 1 | 1 | 1 | 1 | 1 | 1 | 1 |
| Isoyama et al, 2014 (3) | 1 | 1 | 1 | 1 | 1 | 1 | 1 | 1 | 1 | 1 | 1 |
| Xiang et al, 2023 (18) | 1 | 1 | 1 | 1 | 1 | 1 | 1 | 1 | 1 | 1 | 1 |
| Correa et al, 2023 (13) | 1 | 1 | 1 | 1 | 1 | 1 | 1 | 1 | 1 | 1 | 1 |
| Ishimura et al, 2021 (14) | 1 | 1 | 1 | 1 | 1 | 1 | 1 | 1 | 1 | 1 | 3 |
| Xu et al, 2020 (4) | 1 | 1 | 1 | 1 | 1 | 1 | 1 | 1 | 1 | 1 | 2 |
| Sanchez-Tocina et al, 2022 (15) | 1 | 1 | 1 | 1 | 1 | 1 | 1 | 1 | 1 | 3 | 1 |
| Fantinel Ferreira et al, 2022 (17) | 1 | 1 | 1 | 1 | 1 | 1 | 1 | 1 | 1 | 1 | 1 |
| Zhang et al, 2022 (16) | 1 | 1 | 1 | 1 | 1 | 1 | 1 | 1 | 1 | 1 | 1 |
| Sabatino et al, 2024 (6) | 1 | 1 | 1 | 1 | 1 | 1 | 1 | 1 | 1 | 1 | 1 |

**Legend.** The first column refers to study number. Each color represents a level of risk of bias: white boxes, low risk of bias; light grey boxes, unclear risk of bias; black boxes, high risk of bias; dark grey boxes, not-applicable. Questions, Critical Appraisal tools for use in JBI Systematic Reviews: 1. Were the two groups similar and recruited from the same population? 2. Were the exposures measured similarly to assign people to both exposed and unexposed groups? 3. Was the exposure measured in a valid and reliable way? 4. Were confounding factors identified? 5. Were strategies to deal with confounding factors stated? 6. Were the groups/participants free of the outcome at the start of the study? 7.Were the outcomes measured in a valid and reliable way? 8. Was the follow up time reported and sufficient to be long enough for outcomes to occur? 9. Was follow up complete, and if not, were the reasons to loss to follow up described and explored? 10. Were strategies to address incomplete follow up utilized? 11. Was appropriate statistical analysis used?

**Reference of Supplementary tables 3A-C and supplementary figure 1.**

1. Giglio J, Kamimura MA, Lamarca F, Rodrigues J, Santin F, Avesani CM. Association of Sarcopenia With Nutritional Parameters, Quality of Life, Hospitalization, and Mortality Rates of Elderly Patients on Hemodialysis. J Ren Nutr. 2018;28(3):197-207.
2. Yamamoto S, Matsuzawa R, Hoshi K, Suzuki Y, Harada M, Watanabe T, et al. Modified Creatinine Index and Clinical Outcomes of Hemodialysis Patients: An Indicator of Sarcopenia? J Ren Nutr. 2021;31(4):370-9.
3. Isoyama N, Qureshi AR, Avesani CM, Lindholm B, Bàràny P, Heimbürger O, et al. Comparative associations of muscle mass and muscle strength with mortality in dialysis patients. Clin J Am Soc Nephrol 2014; 9: 1720-1728.
4. Xu X, Yang Z, Ma T, Li Z, Chen Y, Zheng Y, Dong J. The cut-off values of handgrip strength and lean mass index for sarcopenia among patients on peritoneal dialysis. Nutr Metabol 2020; 17: 84.
5. Kim JK, Kim SG, Oh JE, Lee YK, Noh JW, Kim HJ, et al. Impact of sarcopenia on long-term mortality and cardiovascular events in patients undergoing hemodialysis. Korean J Intern Med. 2019;34(3):599-607.
6. Sabatino A, Kooman J, Avesani CM, Gregorini M, Bianchi S, Regolisti G, Fiaccadori E. Sarcopenia diagnosed by ultrasound -assessed quadriceps muscle thickness and handgrip strength predicts mortality in patients on hemodialysis. J Nephrol 2024; 37: 993-1003.
7. Souweine JS, Pasquier G, Kuster N, Rodriguez A, Patrier L, Morena M, et al. Dynapaenia and sarcopaenia in chronic haemodialysis patients: do muscle weakness and atrophy similarly influence poor outcome? Nephrol Dial Transplant. 2021;36(10):1908-18.
8. Elder M, Moonen A, Crowther S, Aleksova J, Center J, Elder GJ. Chronic kidney disease -related sarcopenia as a prognostic indicator in elderly haemodialysis patients. BMC Nephrol 2023; 24: 138.
9. Kittiskulnam P, Chertow GM, Carrero JJ, Delgado C, Kaysen GA, Johansen KL. Sarcopenia and its individual criteria are associated, in part, with mortality among patients on hemodialysis. Kidney Int. 2017;92(1):238-47.
10. Mori K, Nishide K, Okuno S, Shoji T, Emoto M, Tsuda A, et al. Impact of diabetes on sarcopenia and mortality in patients undergoing hemodialysis. BMC Nephrol. 2019;20(1):105.
11. Lin YL, Liou HH, Wang CH, Lai YH, Kuo CH, Chen SY, et al. Impact of sarcopenia and its diagnostic criteria on hospitalization and mortality in chronic hemodialysis patients: A 3-year longitudinal study. J Formos Med Assoc. 2020;119(7):1219-29.
12. Song YR, Kim JK, Lee HS, Kim SG, Choi EK. Serum levels of protein carbonyl, a marker of oxidative stress, are associated with overhydration, sarcopenia and mortality in hemodialysis patients. BMC Nephrol. 2020;21(1):281.
13. De Luca Correa H, Bonadias Gadelha A, Vainshelboim B, Tiradentes Dutra M, Ferreira-Junior JB, Alves Deus L et al. Could sarcopenia-related mortality in end-stage renal disease be underpinned by the number of hospitalizations and cardiovascular disease? Int Urol Nephrol 2023; 55: 157-163.
14. Ishimura E, Okuno S, Nakatani S, Mori K, Miyawaki J, Okazaki H et al. Significant association of diabetes with mortality in chronic hemodialysis patients, independent of the presence of obesity, sarcopenia and sarcopenic obesity. J Ren Nutr 2022; 32: 94-101.
15. Sánchez-Tocino ML, Miranda-Serrano B, López-Gonzalez A, Villoria-González S, Pereira-García M, Gracia-Iguacel C et al. Sarcopenia and mortality in older hemodialysis patients. Nutrients 2022; 14: 2354.
16. Zhang M, Zhang L, Hu Y, Wang Y, Xu S, Xie X, et al. Sarcopenia and echocardiographic parameters for predicton of cardiovascular events and mortality in patients undergoing maintenance hemodialysis. PeerJ. 2022;10:e14429.
17. Fantinel Ferreira M, Böhlke M, Belem Pauletto M, Frühauf IR, Gonzalez MC. Sarcopenia diagnosed using different criteria as a predictor of early mortality in patients undergoing hemodialysis. Nutrition 2022; 95: 111542.
18. Xiang T, Fu P, Zhou L. Sarcopernia and osteosarcopenia among patients undergoing hemodialysis. Front Endocrinol 2023; 14: 1181139.
19. Ren H, Gong D, Jia F, Xu B, Liu Z. Sarcopenia in patients undergoing maintenance hemodialysis: incidence rate, risk factors and its effect on survival risk. Ren Fail. 2016;38(3):364-71.

**Supplementary figure 2**. Galbraith plot of studies investigating the effect of confirmed sarcopenia included in the meta-analysis.


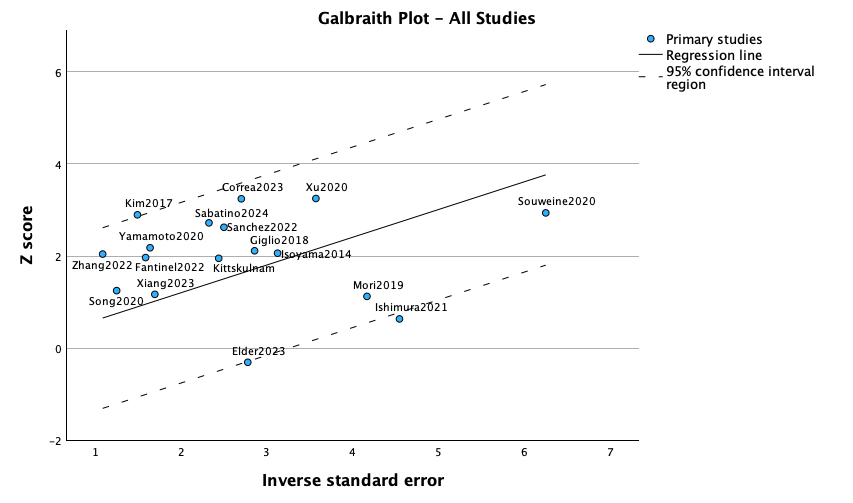


Supplementary figure 3. Funnel plot for the evaluation of publication bias for studies reporting on sarcopenia


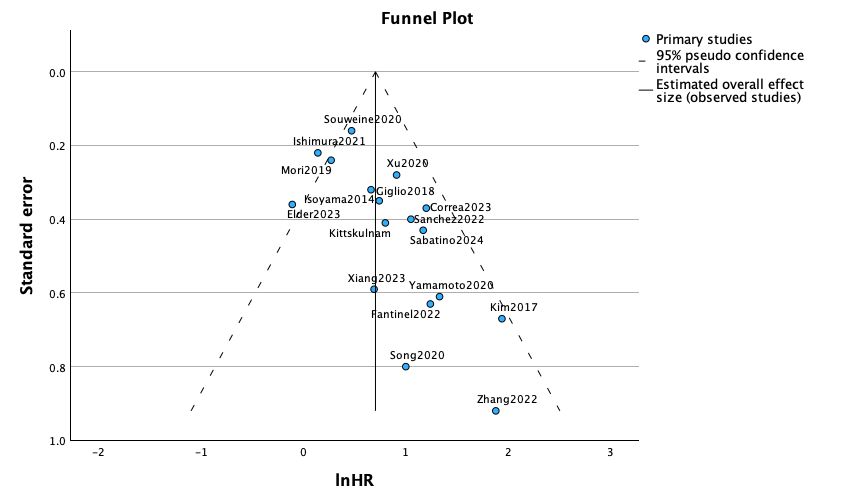


Supplementary Figure 4. Subgroup analysis for the effect of low muscle mass with adequate muscle strength on mortality based on dialysis vintage (incident versus prevalent patients).


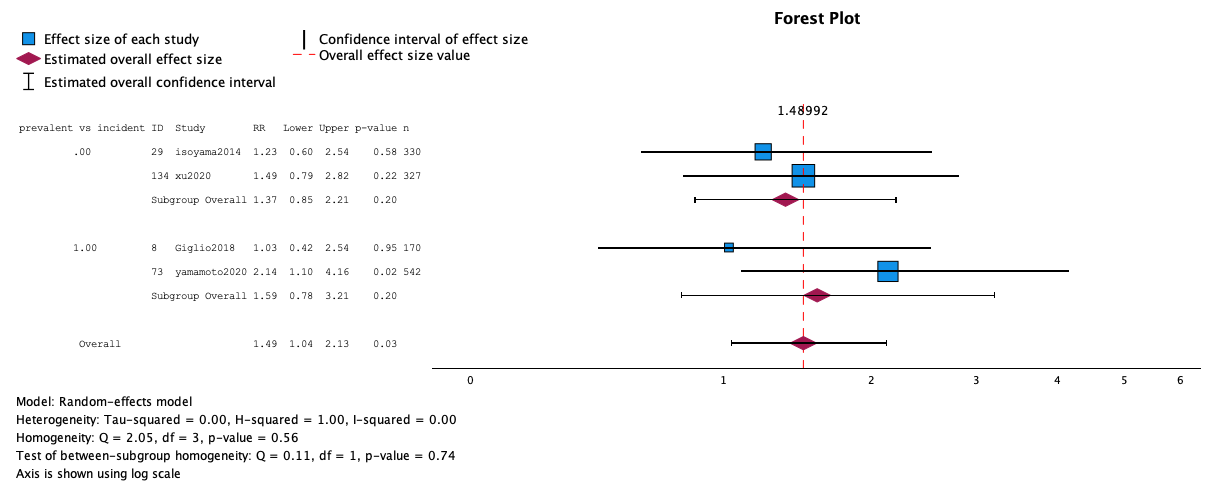


Supplementary Figure 5. Subgroup analysis for the effect of low muscle strength with adequate muscle mass on mortality based on dialysis vintage (incident versus prevalent patients).


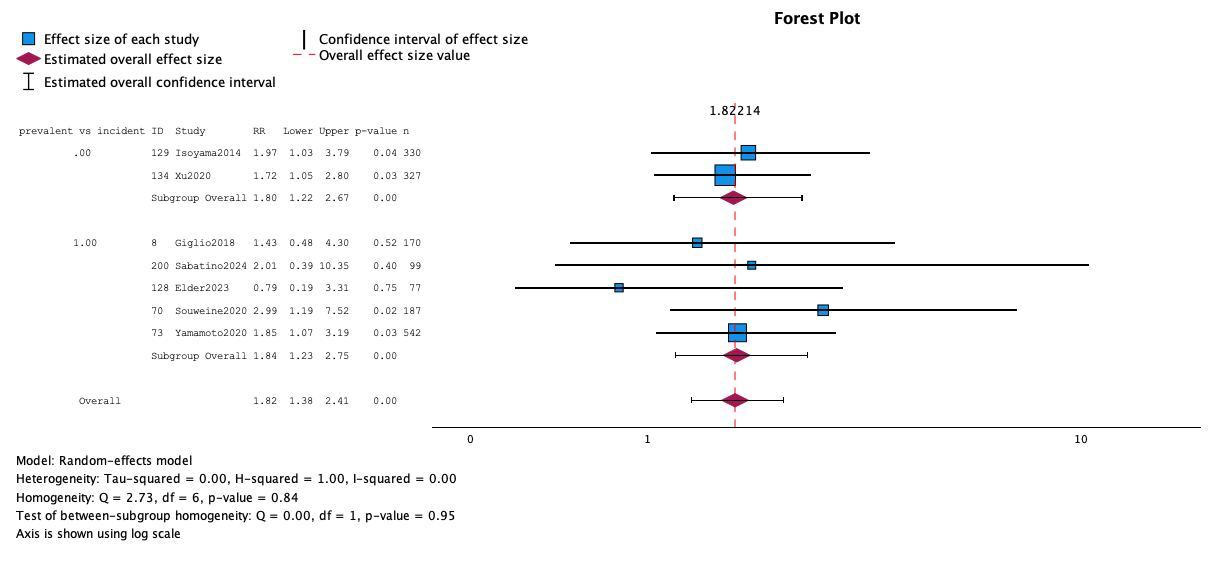


Supplementary Figure 6. Subgroup analysis for the effect of confirmed sarcopenia on mortality based on dialysis vintage (incident versus prevalent patients).


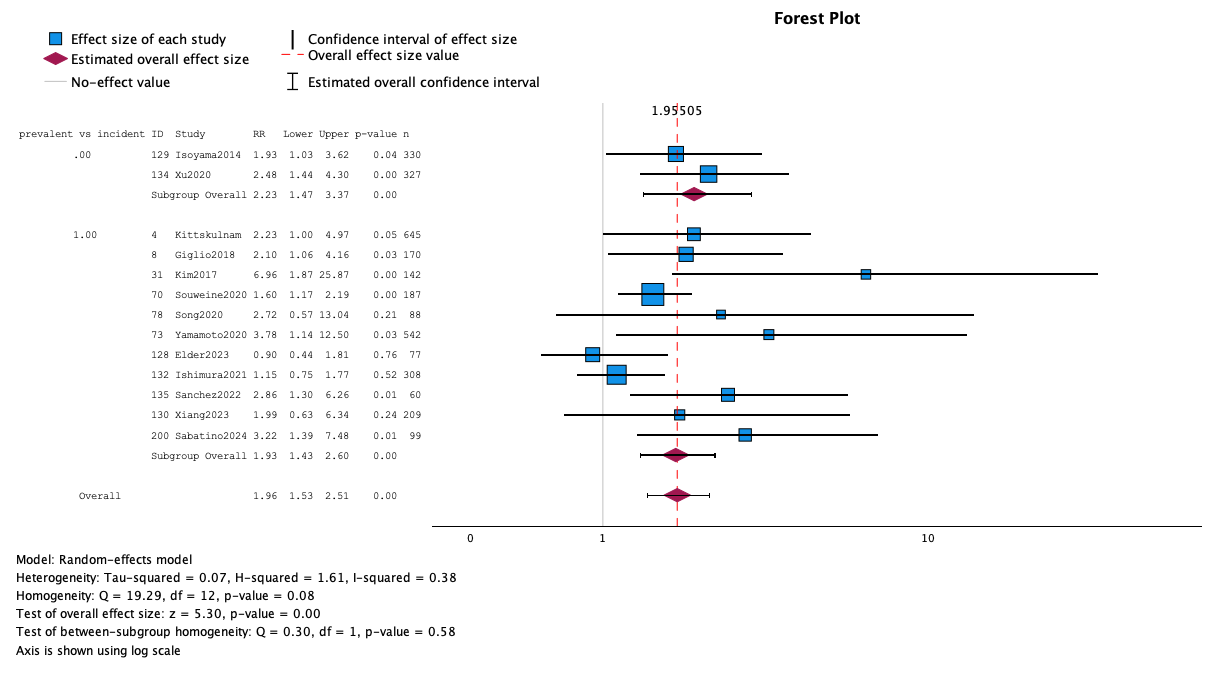


Supplementary Figure 7. Subgroup analysis for the effect of confirmed sarcopenia on mortality based on age (< 60 years versus ≥ 60 years).


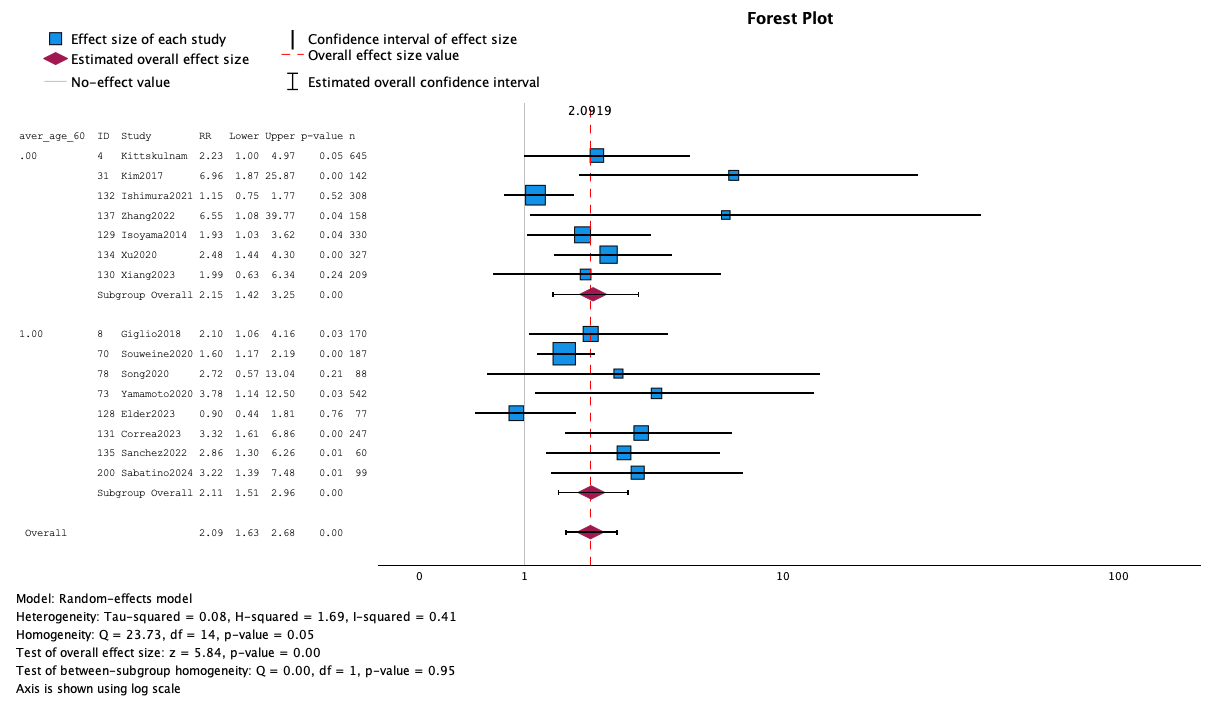


Supplementary figure 8. Subgroup analysis for the effect of confirmed sarcopenia on mortality based on sex (> 50% female versus < 50% female).


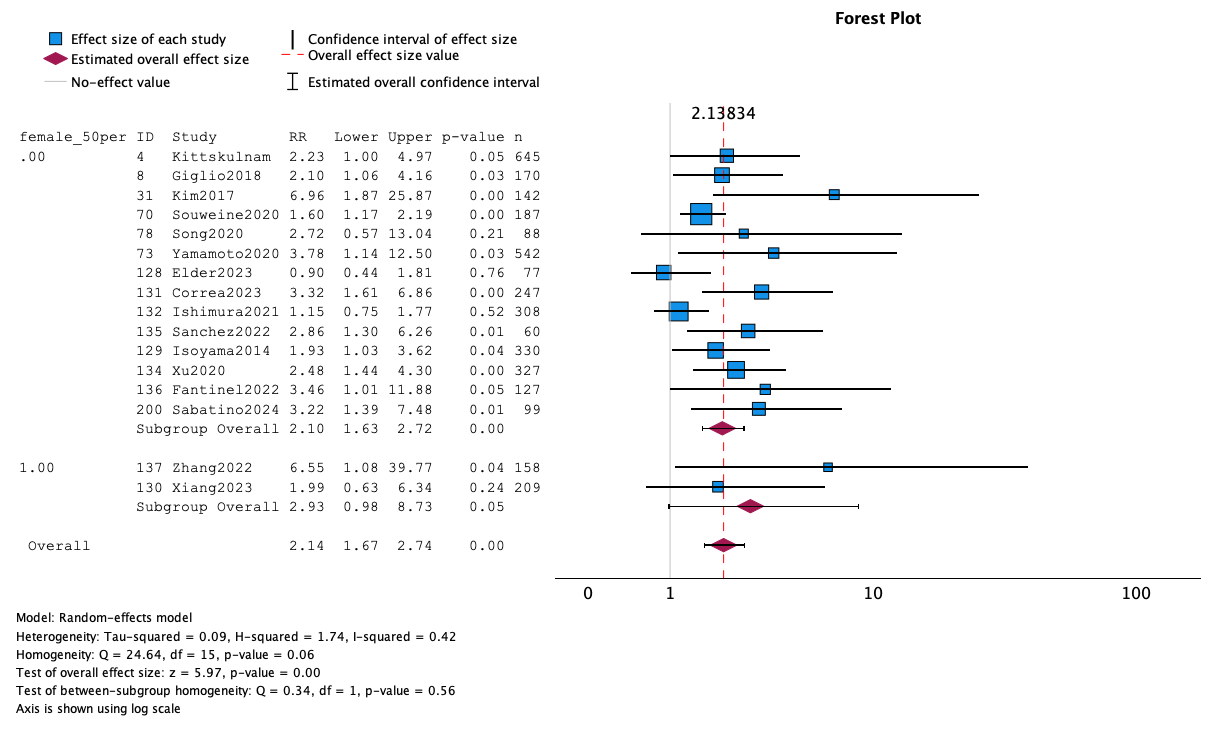

Supplement: Supplementary file 1 — Supplementary Table 1: Eligibility criteria for inclusion of studies in the meta‐analysis and systematic review. Supplementary Table 2: . Detailed search strategy. Supplementary Table 3: Summary of study characteristics and main results separated by measurement of interest (muscle mass, muscle strength and sarcopenia). Supplementary Table 4: Summary of assessment of certainty of evidence (GRADE) for increased risk for mortality. Supplementary Figure 1: Risk of bias. Supplementary Figure 2: Galbraith plot of studies investigating the effect of confirmed sarcopenia included in the meta‐analysis. Supplementary Figure 3: Funnel plot for the evaluation of publication bias for studies on sarcopenia. Supplementary Figure 4: Subgroup analysis for the effect of low muscle mass with adequate muscle strength on mortality based on dialysis vintage (incident vs. prevalent patients). Supplementary Figure 5: Subgroup analysis for the effect of low muscle strength with adequate muscle mass on mortality based on dialysis vintage (incident vs. prevalent patients). Supplementary Figure 6: Subgroup analysis for the effect of confirmed sarcopenia on mortality based on dialysis vintage (incident vs. prevalent patients). Supplementary Figure 7: Subgroup analysis for the effect of confirmed sarcopenia on mortality based on age (< 60 years vs. ≥ 60 years). Supplementary Figure 8: Subgroup analysis for the effect of confirmed sarcopenia on mortality based on sex (> 50% females vs. < 50% females). [file JCSM-16-e70089-s001.docx]
